# GhCDPK60 positively regulates drought stress tolerance in both transgenic *Arabidopsis* and cotton by regulating proline content and ROS level

**DOI:** 10.3389/fpls.2022.1072584

**Published:** 2022-12-01

**Authors:** Mengyuan Yan, Xiaotian Yu, Gen Zhou, Dongli Sun, Yu Hu, Chenjue Huang, Qintao Zheng, Nan Sun, Jiayan Wu, Zhaobin Fu, Libei Li, Zhen Feng, Shuxun Yu

**Affiliations:** ^1^ College of Advanced Agriculture Sciences, Zhejiang A&F University, Hangzhou, China; ^2^ State Key Laboratory of Cotton Biology, Institute of Cotton Research of CAAS, Anyang, Henan, China

**Keywords:** *Gossypium hirsutum* L., *GhCDPK60*, drought stress, reactive oxygen species (ROS), osmotic adjustment (OA)

## Abstract

Calcium-Dependent Protein Kinases (CDPKs) involved in regulating downstream components of calcium signaling pathways play a role in tolerance to abiotic stresses and seed development in plants. However, functions of only a few cotton CDPKs have been clarified at present. In this study, 80 conserved CDPKs in *Gossypium hirsutum* L. were identified and characterized, which was divided into four subgroups. Among them, the transcript level of *GhCDPK60* was significantly upregulated under drought and several hormone treatments. And we found that the expression levels of several stress-inducible genes down-regulated in *GhCDPK60*-silence cotton and up-regulated in *GhCDPK60*-overexpressing *Arabidopsis*. In addition, physiological analyses demonstrated that *GhCDPK60* improved drought stress tolerance by improving the osmotic adjustment ability and reducing the accumulation of reactive oxygen species (ROS) in plants. These findings broaden our understanding of the biological roles of *GhCDPK60* and mechanisms underlying drought stress tolerance in cotton.

## Introduction

Ever deteriorating climatic condition, which has led to significant climate change and exacerbated global warming are detrimental to agricultural production. Moreover, drought is one of the major forms of abiotic stresses, which has led to low water availability for plant utilization thereby negatively affects crop production (Saranga et al., 2009). However, plants have evolved elaborate morpho-physiological and molecular mechanisms to overcome or minimize the growth deficits induced by drought stress, for example, osmotic adjustment, stomatal regulation, photosynthetic response, low leaf water loss, high relative water contents (RWC) or the expression of stress-induced genes (Mahmood et al., 2019). Genetic diversity for drought tolerance in major crops is critical to food security, and a number of genes have been reported to confer tolerance to drought and dehydration stresses in plants, such as the *LEA* (Sun et al., 2021), *CDPK* (Asano et al., 2012a) among others. Drought tolerance is a complex biological process involving multiple genes associated with cellular signaling pathways and molecular responses, such as Mitogen-activated-protein-kinase (MAPK) participating in stress signaling and activate several stress-responsive proteins (Group et al., 2002), calcium (Ca^2+^) responding to drought stress and various hormones (Liu et al., 2014), as well as reactive oxygen species (ROS) triggering defense mechanism (Fang and Xiong, 2015). Among these pathways, calcium is an important regulator and second messenger in signal transduction pathways and cellular biochemical processes, concentration of which alters in response to various stimuli, including hormones and drought stresses (Liu et al., 2014). In higher plants, several Ca^2+^ sensors or Ca^2+^-binding proteins could detect transient Ca^2+^ changes and induce downstream responses, including altered protein phosphorylation and gene expression patterns (Asano et al., 2012a). Three major types of Ca^2+^-binding proteins have been identified in plants: calcium-dependent protein kinases (CDPKs), calmodulins (CaMs) and CaM-like proteins (McCormack et al., 2005), as well as calcineurin B-like proteins (Kolukisaoglu et al., 2004). Notably, the CDPKs were widely reported to be involved in detecting and transmitting cellular calcium signals that respond to drought stress (Zou et al., 2010; Asano et al., 2012a; Wei et al., 2014).

CDPKs, a unique family of Ca^2+^ sensor/kinase-effector proteins, are involved in both phosphorylation cascades and Ca^2+^ signaling (Valmonte et al., 2014). CDPKs have a typical domain architecture, with an autoinhibitory junction connecting a Ser/Thr protein kinase domain with an Ca^2+^-binding domain-containing EF-hand, flanked by variable regions on the N-terminal and C-terminal (Klimecka and Muszyńska, 2007). Upon binding Ca^2+^, the CDPKs undergo a conformational change that reveal their active sites, and then tend to autophosphorylation and/or phosphorylate downstream targets, such as ion, transcription factors, and metabolic enzymes (Wernimont et al., 2010; Liese and Romeis, 2013). CDPKs are identified in all land plants (Harmon et al., 2001; Li et al., 2008); there are 34 *CDPKs* genes identified in the *Arabidopsis thaliana* (Cheng et al., 2002; Hrabak et al., 2003), 31 genes in the *Oryza sativa* (Asano et al., 2005), and 30 genes in the *Populus trichocarpa* (Zuo et al., 2013). Increasing reports have evidenced the involvement of different CDPKs in plant abiotic/biotic stress and development responses. Overexpression of *AtCDPK1* significantly enhanced the resistance to salt or drought stress in *Arabidopsis* (Huang et al., 2018). AtCDPK4 and AtCDPK11 are two important positive regulators in ABA signaling pathways and salt stress (Zhu et al., 2007). AtCDPK8 and AtCDPK10 function in ABA- and Ca^2+^-mediated plant responses to drought stress through phosphorylating downstream genes (Zou et al., 2010; Zou et al., 2015). AtCDPK23 plays a negative part in plant response to drought and salt stress (Ma and Wu, 2007), but AtCDPK27 is required for plant adaptation to salt stress (Zhao et al., 2015). In addition to abiotic stress, several *Arabidopsis* CDPKs have been verified to participate in the plant innate immune response, such as AtCDPK1, AtCDPK5 (Coca and San Segundo, 2010; Liu et al., 2017). In rice, OsCPK9, OsCDPK12 and OsCDPK21 modulate the ABA signaling pathway and salt stress responses (Asano et al., 2012b; Wei et al., 2014; Chen et al., 2017). The maize ZmCDPK1 plays a negative role in cold stress signaling (Weckwerth et al., 2015), while ZmCDPK4 positively regulates ABA signaling and enhanced drought stress tolerance (Jiang et al., 2013). Together, these studies indicate that CDPK-mediated abiotic stress and ABA responses are complex and conserved in plants.

By contrast, only a few *CDPKs* have been characterized in cotton. Cotton (*Gossypium hirsutum* L.) is the staple source of fiber worldwide and the best crop of polyploidization study (Li et al., 2015a). Nevertheless, cotton yields and fiber quality have been adversely affected by climate change features, such as drought (Li et al., 2019). Therefore, improvement of cotton drought tolerance could reduce drought-induced yield loss and enable the expansion of cotton cultivation. Previous researchers have demonstrated that plants can sense and respond to abiotic stresses through various functional proteins. In previous study, 41 *CDPKs* genes were identified after sequencing of the *Gossypium raimondii* genome, but functional analysis were not performed (Li et al., 2015b). In upland cotton, which accounts for more than 90% of commercial cotton production worldwide, only a few *GhCDPKs* have been characterized. GhCDPK1 is involved in cotton fiber growth regulation by phosphorylating GhACS2 (Wang et al., 2011). Prior to now, however, functional studies of the *CDPK* family in upland cotton, particularly in response to drought stress, have not been properly focused. In this study, we identified 80 *GhCDPK* genes from upland cotton genomes and performed bioinformatics analysis to understand the structure of GhCDPKs, along with sequence similarity, and finally chose *GhCDPK60* that linked to drought stress tolerance for further studies. The *GhCDPK60* showed significantly upregulated expression under drought and several hormone treatments. To get insight into the function of *GhCDPK60* in regulating drought tolerance in cotton, we carried out the functional characterization through overexpression and knockdown of *GhCDPK60* in *Arabidopsis* and cotton, respectively. Our study revealed that *GhCDPK60* as a candidate for cotton genetic improvement and provided insights into cotton’s drought stress tolerance mechanisms.

## Materials and methods

### Plant materials, growth conditions, and treatments

Seeds of ‘TM-1’ and ‘CRI50’ were used in current investigation. TM-1 is widely used as a genetic standard (Zhang et al., 2015), and CRI50 is a commercial Chinese cotton cultivar with high yield and stress tolerance (Li et al., 2021). Cotton seeds were delinted with H_2_SO_4_ (98%) and rinsed in water, then soaked in distilled water for 1 day and followed by germination on wet gauzes for another day at 25°C. *Nicotiana benthamiana* RA-4 accession was used for subcellular location experiment. Germinant cotton seeds and *Nicotiana benthamiana* were transferred to pots containing vermiculite in the greenhouse at 28/20°C under 16-h light/8-h dark photoperiod (60% relative humidity). *Arabidopsis thaliana* ecotype Columbia-0 (Col-0) was used for transgenic analysis. The *Arabidopsis thaliana* and all transgenic plants were planted in the growth chamber at a temperature regime of 23/20°C with 16-h light/8-h dark cycle (50% relative humidity).

For drought treatment, ‘TM-1’ were used to water withholding at the trefoil stage of seedlings for two weeks, and rewatering for three days. A no treatment control was always included. Transcript levels were detected in cotton seedling. Hormone treatment were performed at the trefoil stage of the cotton seedlings with solutions of various substances. Cotton seedlings were sprayed evenly on the leaves with ABA solution (100 µM), MeJA solution (100 µM), IAA solution (100 µM), SA solution (100 µM), or GA solution (100 µM) for up to 24 h. The leaf samples were collected at 0, 6, 12, 24 h after treatment to assess candidate gene expression response to various hormone. These samples were frozen in liquid nitrogen immediately and stored at -80°C for RNA isolation. Three biological repeats in each treatment were performed.

### Identification and motif analysis of *GhCDPKs*

34 *AtCDPKs* sequences were downloaded from the *Arabidopsis* Information resource (http://www.arabidopsis.org/), and 30 *OsCDPKs* sequences have been downloaded from either China Rice Data Center (https://www.ricedata.cn/gene/) or GenBank (http://ncbi.nlm.nih.gov/genbank). The *G.hirsutum* genome sequence was downloaded from the sequenced genome in Cottongen (https://www.cottongen.org). These CDPK proteins in model plants were set as the query in a BLASTp (https://blast.ncbi.nlm.nih.gov/Blast.cgi) search to identify the CDPK proteins in *G.hirsutum*. Meanwhile, to identify the GhCDPK family members more accurately, the profile hidden Markov model of the HMMER3.0 program was further applied to search all of the hits with the default parameters by utilizing EF hand domains (PF13499) and kinases (PF00069) (Finn et al., 2011). Candidate genes were obtained by combining two results and removing the duplicates (**Supplementary Table S1**). SMART (http://smart.embl-heidelberg.de/) and PFAM (http://pfam.janelia.org/) were used to verify the presence of protein kinase domains and EF hand domains in all candidates. Motif prediction and visualization of *GhCDPKs* was done by MEME website (http://meme-suit/org/) and MEME Suit Wrapper of TB tools, respectively. The molecular mass of each GhCDPKs were predicted by the online tools ProtParam (http://web.expasy.org/protparam/).

### Chromosomal localization and phylogenetic analysis of *GhCDPKs*

Based on physical location data provided in Cottongen, the chromosomal locations of *GhCDPK*s were predicted and subsequently visualized using Gene Location Visualize from GTF/GFF tool of TBtools software. Multiple amino acid sequence alignment was performed using ClustalW. The phylogenetic tree was constructed by MEGA7.0 using the neighbor-joining method with 1000 bootstrap replications (Kumar et al., 2016) and then displayed with the online iTOL tool (https://itol.embl.de/tree).

### Expression profile analysis of *GhCDPKs*

The expression profile in *G. hirsutum* TM-1 at different tissues, including root, stem, leaf, bract, torus, pental, pistil, filament, anther, ovules and fibers, were obtained from published RNA-seq dataset reported previously (Zhang et al., 2015). The expression data were gene-wise normalized, and the expression patterns were illustrated using the MultiExperiment Viewer (MeV) software.

### RNA isolation and quantitative real-time PCR

Total RNA was extracted from the frozen samples with three replicates using the RNA Prep Pure kit (TIANGEN Biotech) following the manufacture’s recommendations. The first-strand cDNA was synthesized from 2 μg total RNA based on a PrimeScript Reverse Transcriptase Kit (Takara). qRT-PCR was performed in a LightCycler 480 II PCR System (Mannheim, Germany) using the SYBR Premix Ex Taq (Takara) with *GhACT4* (Artico et al., 2010) and *AtUBQ7* (Zhang et al., 2021b) as reference genes in cotton and *Arabidopsis*, respectively. Relative quantification in gene expression levels were calculated based on three biological replicates referring the 2^-ΔΔCt^ method (Livak and Schmittgen, 2001). Specific primers used for qRT-PCR are listed in **Supplementary Table S2**.

### Physicochemical properties analysis and subcellular location of *GhCDPK60*

The physicochemical property analysis of GhCDPK60 were predicted by the online tools CBS (http://www.cbs.dtu.dk/services/). Cis-regulatory elements in the promoter of *GhCDPK60* were predicted through online website plantCARE (http://bioinformatics.psb.ugent.be/webtools/plantcare/html/). Subcellular location prediction of GhCDPK60 was carried out by using the WoLF PSORT website (https://wolfpsort.hgc.jp/). In order to detect the subcellular localization of *GhCDPK60 in vivo*, we used tobacco for transient expression. The full-length coding region of *GhCDPK60* was amplified from the *G. hirsutum* variety TM-1 and cloned into the pCAMBIA1305-GFP vector driven by *CaMY35S* promoter to generate GFP-fusion protein. Primer pairs were listed in **Supplementary Table S2**. Subsequently, the plasmids were introduced into *Agrobacterium tumefaciens* strain GV3101 and then infiltrated 3-4-week-old *Nicotiana benthamiana* leaves epidermis. The signal of GFP was observed after 48 h using a laser confocal scanning microscope (LSM 880, Zeiss, Germany). The 35S-OsAlaAT1-mCherry (Yang et al., 2015; Fang et al., 2022) and 35S-mCherry-SYP132 (Xia et al., 2019) were used as plant cytoplasm and plasma membrane marker for the colocalization experiments, respectively. Excitation wavelength used in 488 nm for GFP, and the wavelength range of captured light at 515-555 nm. The excitation wavelength and gain wavelength of mCherry were 555 nm and 580-630 nm, respectively.

### Generation and analysis of transgenic *Arabidopsis*

The coding sequence of *GhCDPK60* was amplified and cloned into a pBI121 vector driven by *CaMY35S* promoter. Primer pairs were listed in **Supplementary Table S2**. The recombinant plasmid was transformed into *Arabidopsis thaliana* (Columbia-0, Col-0) through the floral dipping method (Clough and Bent, 1998), and positive transgenic plants were selected on the 1/2 Murashige and Skoog (MS) medium containing 50 mg/mL kanamycin. Gene-specific primers were used to isolate homozygous plants and confirm transcription status. T_3_ transgenic pure lines were subjected to gene expression analysis and stress tolerance evaluation.

To observe the effects of ABA and mannitol on seed germination and phenotypic differences between wild type and *OE*-*GhCDPK60* plants, three independent *GhCDPK60*-overexpressing lines (T_3_) and Col-0 plants were tested according to method mentioned previously (Wang et al., 2016). Col-0 and *GhCDPK60*-overexpressing seeds were sown on 1/2 MS medium solid plates with 0.25 μM ABA, 200 mM or 400 mM mannitol, stratified at 4°C for 3 days and then transferred to long-day growth conditions (16-h light/8-h dark cycle at 25°C) in the growth chamber.

To identify the stress tolerance of *GhCDPK60* overexpressed *Arabidopsis* (OE4, OE5 and OE12) and Col-0, seeds cultured on MS medium for 7 days were transplanted into soil for about 3 weeks with sufficient watering followed by a 15-days drought stress (withholding irrigation). Normally watered plants were used as the control. And then the relative water content, malondialdehyde (MDA), proline, catalase (CAT) activities, superoxide dismutase (SOD) activities, and peroxidase (POD) activities were examined by leaf samplings. Each sample represented three replicates (each replicate had 4-6 seedlings). These experiments were performed at least three times independently with similar results.

### VIGS assay in cotton and phenotypic profiling under drought stress

An about 360 bp fragment of *GhCDPK60* was amplified and then ligated into Virus Induced Gene Silencing (VIGS) vector pTRV2 (Reyes et al., 2017). Constructed vectors were transformed into Agrobacterium GV3101 competent cells by a heat-shock method. The GV3101 lines contained pTRV-*GhCDPK60*, pTRV-*GhPDS*, and pTRV (pYL156, empty vector as control) were mixed with an equal volume of Agrobacterium containing pYL192 (helper vector), respectively. The mixed solutions were used to infiltrate unfolded cotton cotyledons of TM-1 and CRI50, respectively (Hayward et al., 2011; Gu et al., 2014). The VIGS experiments were repeated at least three times with more than three individual plants were included. The quantitative real-time PCR (qRT-PCR) was performed to further confirm that candidate genes had been silenced in VIGS experiments. The primers used in the VIGS experiments and qRT-PCR analysis are listed in **Supplementary Table S2**.

For drought tolerance assay, when pTRV-*GhPDS* plants showed phenotype, pTRV-*GhCDPK60* and pTRV plants were subjected to water withholding at the trefoil stage of seedlings. The phenotypes were observed after drought treatment for two weeks, and the leaf samples were collected to evaluate relative water content, contents of MDA, proline, activities of CAT, SOD, and POD. The treatments were repeated at least twice.

### ROS dyeing

In order to observe the accumulation of H_2_O_2_, fresh leaves from control and VIGS-cotton after two-weeks drought stress treatment were taken and incubated completely in 1mg/mL, pH 3.8 3-3’-diaminobenzidine (DAB) solution for 6 h under 70% humidity conditions till brown precipitates are observed, and then decolorized in 96% ethanol at 40°C in order to remove chlorophyll (Jambunathan, 2010). For the ${\text{O}}_{2}^{-}$ detection, detached leaves were immersed in 100 mL staining solution containing 0.1% (w/v) nitroblue tetrazolium (NBT), 10 mM sodium azide, 50 mM potassium phosphate, pH 6.4 for 15 min. After stopping the reaction with 95% ethanol, the samples were decolorized in 96% ethanol under heating at 40°C (Jambunathan, 2010). Superoxide ions react with NBT and appear as blue. These stained leaves can be photographed by light microscope.

### Physiological indices measurements

To check the physiological indices changes, the gene-silenced cottons leaves or overexpressing *Arabidopsis* seedlings were used to identify relative water content (RWC), contents of MDA, proline, activities of CAT, SOD, or POD after drought treatments, whereas some plants were kept untreated as controls. Relative water content was measured according to (Hu et al., 2013). The dehydrated leaves were soaked in distilled water for 4 h and turgid weight (TW) was recorded. Leaves were finally dried for 48 h at 80°C to obtain total dry weight (DW). Relative water content was calculated as follows: RWC (%) = [(desiccated weight – DW)/(TW– DW)] × 100. The MDA concentration was determined through thiobarbituric acid method described by (Schmedes and Hølmer, 1989), and proline content measured *via* a ninhydrine method (Irigoyen et al., 1992). The CAT activity, SOD activity, and POD activities were determined according to the previous studies (Alici and Arabaci, 2016; Ullah et al., 2018). The absorbance was measured using a UV-2550 UV-vis spectrophotometer (SHIMADZU). Three biological replications were performed.

## Results

### Identification and phylogenetic tree analysis of the *GhCDPKs* gene family

**Figure 1:**
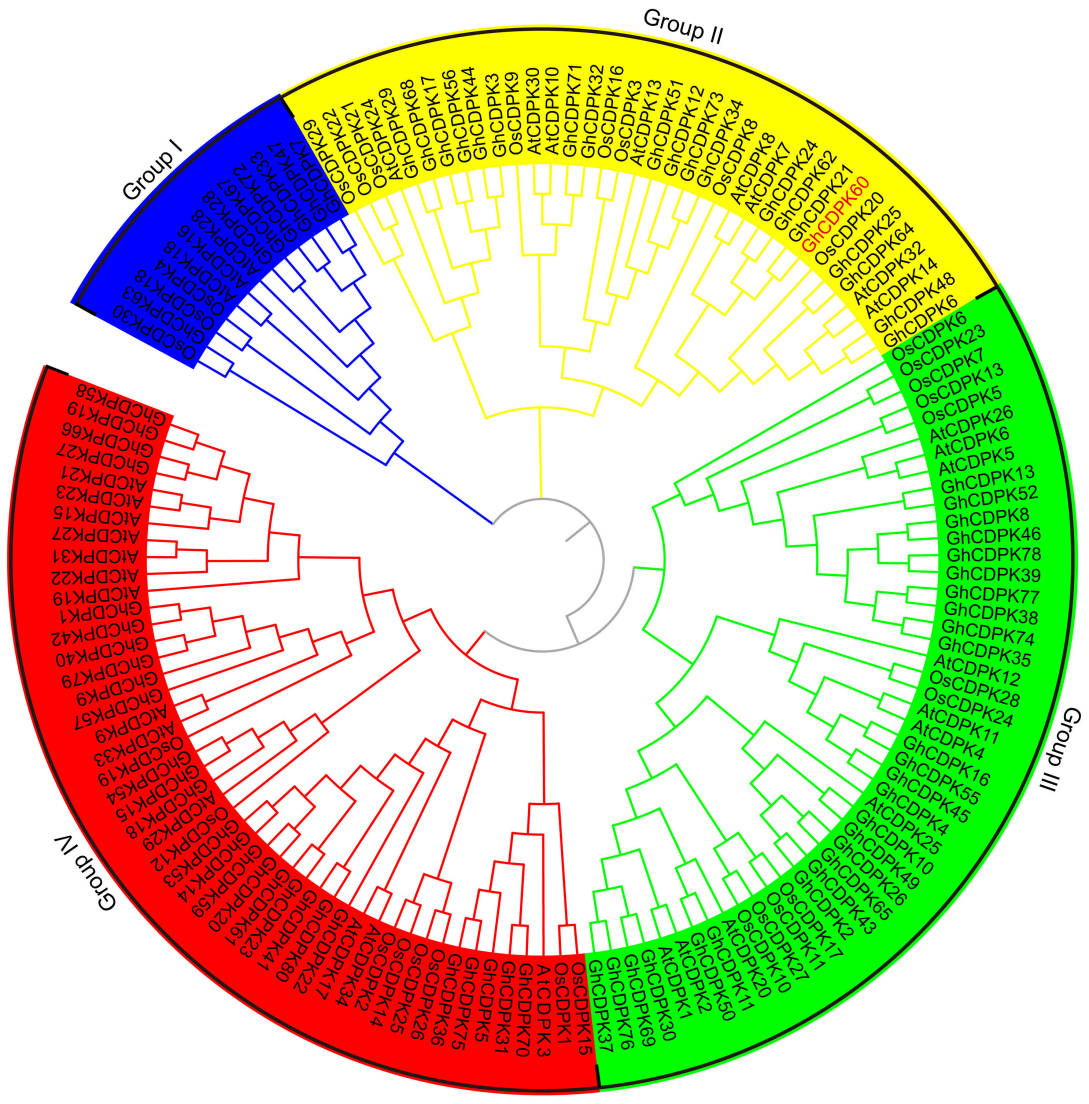
Phylogenetic analysis of predicted CDPK proteins from three different plants species. The phylogenetic tree was generated from the alignment result of the full-length amino acid sequences by the neighbor-joining (NJ) method. All CDPKs members, together with homologs of *Arabidopsis* and rice, were classified into four distinct clades shown in different colors. The prefixes At, Os and Gh are used to identify CDPK proteins from *A. thaliana*, *O. sativa* and *G. hirsutum*, respectively.

To determine the *GhCDPK* genes in *G. hirsutum*, we performed a genome-wide prediction of *GhCDPK* genes by BLAST analysis of *Arabidopsis* and rice CDPKs against the publicly available *G. hirsutum* genome (https://www.cottongen.org). Meanwhile, we conducted a hidden Markov model profile-based search for putative CDPK amino acid sequences and then identified probable *GhCDPKs* using SMART and PFAM. A total of 80 *GhCDPKs* were identified and numbered according to their genome location (**Supplementary Table S1**, **Supplementary Figure 1**). All putative *GhCDPKs* exhibited the typical protein structures of the CDPK family, which consist of a variable N-terminal domain, a protein kinase domain, a junction domain, a calmodulin-like Ca^2+^ binding domain, and a C-terminal domain (**Supplementary Figure 2**). The number of amino acid residues, molecular mass, and isoelectric point of the *GhCDPKs* are listed in **Supplementary Table S1**. The numbers of GhCDPKs amino acids ranged from 368 (GhCDPK51) to 648 residues (GhCDPK11), the predicted molecular mass varied from 41.626 (GhCDPK51) to 63.564 kDa (GhCDPK11) and the predicted isoelectric point ranged between 4.452 (GhCDPK49) and 9.481 (GhCDPK72), which were comparable with *CDPK* genes from other plant species (Hrabak et al., 2003; Ray et al., 2007; Ma et al., 2013). To investigate the evolutionary relationships of the GhCDPKs proteins, sequences of the 80 GhCDPKs, *Arabidopsis* AtCDPKs proteins (Hrabak et al., 2003), and rice OsCDPKs proteins (Ray et al., 2007) were used to construct a neighbor-joining (NJ) phylogenetic tree (**Figure 1**). According to evolutionary relationships, all the CDPKs can be classified into four groups (Group I-IV). CDPKs from monocots and dicots populated evenly in all four groups, which indicated that the CDPKs have already diverged from the common ancestor of these clades. CDPKs were reported to play essential roles in tolerance to abiotic stresses or seed development in plants (Coca and San Segundo, 2010; Asano et al., 2012b; Wei et al., 2014; Liu et al., 2017; Chen et al., 2017). We used public available high-throughput sequencing data from *G. hirsutum* acc. TM-1 to investigate the expression profiles of CDPK family genes in various tissues, including roots, stems, leaves, -3 days post anthesis (dpa), 0 dpa and 3 dpa ovules and 5 dpa, 10 dpa, 20 dpa and 25 dpa fibers among others (Zhang et al., 2015). The *CDPK* genes exhibited various expression patterns and functional divergence in vegetative and reproductive organs (**Supplementary Figure 3**). In our previous genome-wide analysis of the CDPK family in *Gossypium raimondii*, we identified some *CDPK* genes as positive regulators in the response of cotton to drought stress, including *GhCDPK60* (Zhu et al., 2013; Li et al., 2015b). GhCDPK60 shares a high sequence similarity with *Arabidopsis* AtCDPK8 (79.1% identity) (Zou et al., 2015) and both belongs to group II of the CDPK family (**Figure 1**), suggesting their functional similarity; thus, GhCDPK60 was further used for functional study.

### *GhCDPK60* expression is upregulated in response to drought stress and hormonal signal in cotton

**Figure 2:**
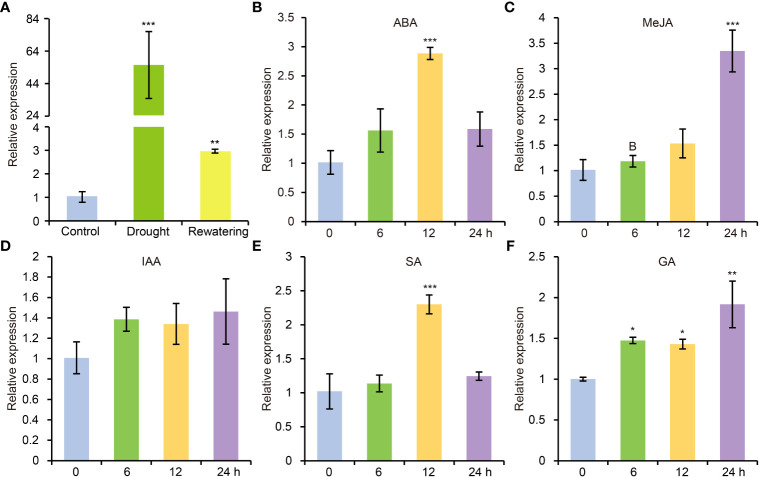
Expression analysis of *GhCDPK60 via* qRT-PCR after drought and hormone treatments. **(A)** Transcription level of *GhCDPK60* after drought stress and rewatering treatments. **(B-F)** Expression patterns of *GhCDPK60* in leaves of TM-1 seedlings sprayed with ABA, MeJA, IAA, SA and GA. Values represent the mean ± SE from three biological replicates. Statistically significant differences compared to 0 h (not-treated control). *P< 0.05, **P< 0.01 and ***P< 0.001 by Student’s *t* test.

According to previous study, AtCDPK8 functions in ABA-mediated stomata regulation in response to drought stress through phosphorylate AtCAT3 (Zou et al., 2015). In view of high similarity of protein sequence between GhCDPK60 and AtCDPK8, we deduced that GhCDPK60 maybe participate in drought stress tolerance. To verify the hypothesis, we carried out drought treatment and found that transcription level of *GhCDPK60* was significantly induced after water withdrawing but recovered to comparably lower after rewatering treatment (**Figure 2A**). Phytohormones play crucial roles in mitigating and minimizing drought stress-related detrimental effects and improving plant growth and survival under different environmental stresses (Jogawat et al., 2021). In order to investigate whether *GhCDPK60* respond to hormonal signals, we evaluated the transcript profiles of *GhCDPK60* according to different hormonal treatments for trefoil stage of the cotton seedlings. Results showed that *GhCDPK60* was upregulated after four types of hormone treatments (**Figure 2**). During the ABA treatment, the expression level of *GhCDPK60* was at least two-fold higher than that of the control within 12 h (**Figure 2B**). The expression level of *GhCDPK60* was approximately three-fold higher than that of the control after 24 h of MeJA treatment (**Figure 2C**). In addition, the expression level of *GhCDPK60* was approximately two-fold higher than that of the control after 12 h of treatment with SA (**Figure 2E**). The distinct expression patterns of *GhCDPK60* suggested that it could respond to drought stress and might function differently during different drought processes.

### GhCDPK60 is a plasma membrane protein

**Figure 3:**
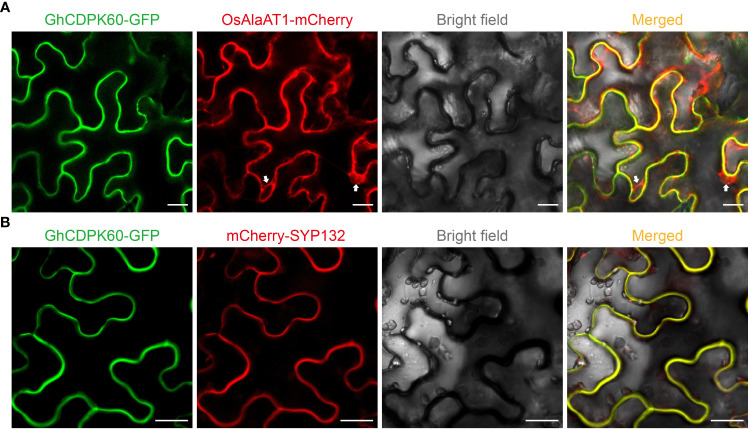
Subcellular localization of GhCDPK60. GhCDPK60 were transiently co-expressed with different marker, OsAlaAT1 **(A)** or SYP132 **(B)**, in *N. benthamiana* leaf cells to determine its subcellular localization. White allows indicate the signals of cytoplasm component. Bars = 20 µm.

The *GhCDPK60* (*Ghir_D07G013310.1*) has an open reading frame of 1590 bp encoding a 59.438 kDa protein, and possesses a structure typical of the CDPK family. In addition, there were several cis-regulatory elements in the promoter of *GhCDPK60* through online website analysis (plantCARE), such as ABRE, Box 4, CAAT-box, CGTCA-motif, G-box, TATA-box, and MBS among others (**Supplementary Table S3**), which are the binding sites for abiotic stress factors, indicating that this gene is essential to stress responses. CDPKs were predicted to localize on the plasma membrane, cytoplasm, endoplasmic reticulum, as well as in the nucleus (Asano et al., 2012a). To confirm subcellular localization of GhCDPK60, the fusion protein of GhCDPK60 with GFP under the constitutive *CaMV35S* promoter was successfully expressed in the epidermal cells of tobacco, and the free GFP vector was used as a positive control (**Supplementary Figure 4**). Transient expression in tobacco leaves was performed by agroinfiltration method. The empty GFP protein was localized in the membrane, cytoplasm, and nucleus, while the signal of GhCDPK60-GFP was perceived in the plasma membrane (**Supplementary Figure 4**). To further confirm the plasma membrane localization of GhCDPK60, GhCDPK60-GFP fusion protein was transiently co-expressed with cytoplasm (**Figure 3A**) and plasma membrane (**Figure 3B**) markers in leaf epidermal cells of *N. benthamiana*, respectively. The results showed that the colocalization signal of GhCDPK60-GFP with plasma membrane marker, SYP132 (Xia et al., 2019), instead of cytoplasm marker were captured (**Figure 3**), indicating that GhCDPK60 is a plasma membrane associated protein kinase.

### The overexpression of *GhCDPK60* improves drought tolerance of *Arabidopsis*

**Figure 4:**
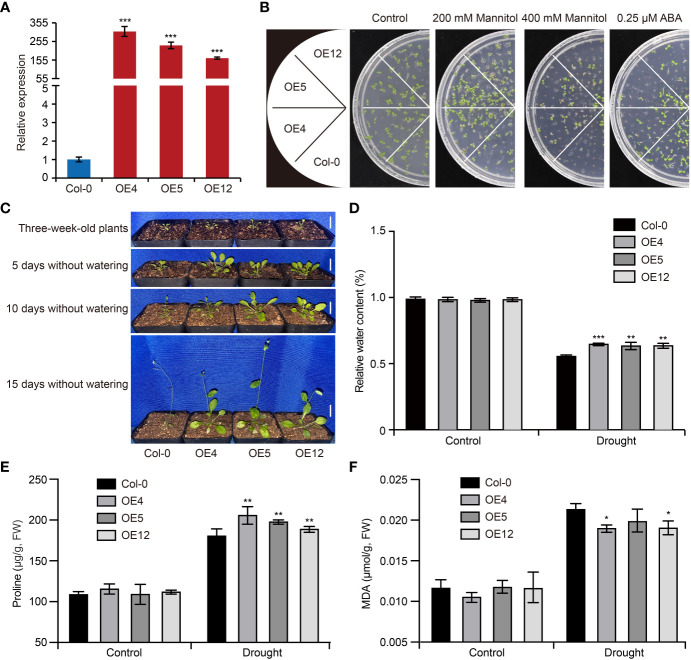
Overexpression of *GhCDPK60* increases drought tolerance in *Arabidopsis*. **(A)** The expression level of overexpressed-*GhCDPK60* in *Arabidopsis*. Col-0, wild-type *Arabidopsis*; OE4, OE5 and OE12, three independent lines. **(B)** Seed germination of Col-0 and *GhCDPK60*-overexpressing *Arabidopsis* under drought stress and ABA treatments. Seeds were germinated on 1/2 MS agar plates with or without ABA and mannitol. Photographs were taken 10 days after 0.25 μM ABA, 200 mM or 400 mM mannitol treatments. **(C)** Phenotypes of Col-0 and transgenic lines after drought treatment. Three-week-old seedlings were deprived of water for 5, 10 or 15 days. Bars = 1 cm. **(D)** Relative water content of Col-0 and transgenic lines with or without drought treatment for 15 days. **(E, F)** The analysis of proline **(E)** and MDA **(F)** concentrations of leaves in Col-0 and *GhCDPK60*-overexpressing lines after drought stress treatment. Statistically significant differences compared to Col-0. Values represent the mean ± SE from three biological replicates. *P< 0.05, **P< 0.01 and ***P< 0.001 by Student’s *t* test.

To confirm the function of *GhCDPK60* in response to drought stress, we firstly overexpressed it in wild-type *Arabidopsis* and selected three independent homozygous lines of the T_3_ generation (OE4, OE5 and OE12) *via* qRT-PCR (**Figure 4A**), which were used for the subsequent physiological experiment. In order to examine whether *GhCDPK60* affects drought tolerance in transgenic *Arabidopsis*, we assessed the germination of Col-0 and *GhCDPK60*-overexpressing lines after 200 mM or 400 mM mannitol treatments. The results showed that *GhCDPK60*-overexpressing lines germination was obviously higher than that of Col after mannitol treatment (**Figure 4B**). The phytohormone ABA plays important roles in the adaptation of plants to abiotic stresses, such as high salinity and drought (Cutler et al., 2010). Drought stress induces ABA accumulation and triggers ABA-dependent signaling pathways (Zhu, 2002). Thus, we investigated the *GhCDPK60* response to ABA and found that transgenic *Arabidopsis* exhibited more sensitivity in medium with 0.25 μM ABA (**Figure 4B**), suggesting that *GhCDPK60* may be involved in ABA-regulated physiological processes and drought stress tolerance.

To further investigate the drought stress tolerance of *GhCDPK60*-OE, 3-week-old *Arabidopsis* seedlings were subjected to a drought treatment. After 5, 10 or 15 days of drought treatment, the growth of Col was inhibited compared with that of *GhCDPK60*-OE lines (**Figure 4C**). Plants with high capacity for water retention can better survive drought or dehydration stress. After a dehydration treatment, *GhCDPK60*-OE lines retained a significant high RWC compared with the Col (**Figure 4D**). In plants, osmotic adjustment and stomatal closure are the main physiological mechanisms for reducing water loss under dehydration or drought. To elucidate the physiological mechanism by which *GhCDPK60* confers tolerance to drought and dehydration stresses and improves the ability of plant to retain water, we quantified the osmolyte proline (Per et al., 2017) in *GhCDPK60*-OE lines. Under normal growth conditions, there were no significant differences between controls and transgenic lines in terms of their proline content, but *GhCDPK60*-OE lines accumulated larger amounts of proline under drought conditions (**Figure 4E**). MDA is the product of the peroxidation reaction, which used as a drought indicator to evaluate the degree of plasma membrane damage and the strength of the stress reaction (Zhang et al., 2021a). To assess whether *GhCDPK60* is involved in oxidative damage, we tested the MDA concentration. Although there were no significant differences in malondialdehyde (MDA) contents between controls and *GhCDPK60*-OE lines under normal growth conditions, clear differences were observed between control and *GhCDPK60*-OE lines after drought treatment (**Figure 4F**). These results indicated that *GhCDPK60* played a positive role in drought stress tolerance.

Under drought stress, the transcript levels of stress-responsive genes *AtCAT3*, *AtNXH1*, *AtRD29A/B*, and *AtDREB2A* were obviously higher in *GhCDPK60*-OE lines than in Col (**Supplementary Figure 5**). In previous studies, higher transcript levels of *AtCAT3*, *AtNXH1*, *AtRD29A/B*, and *AtDREB2A* enhanced tolerance to abiotic stresses (Sakuma et al., 2006; Asif et al., 2011; Jia et al., 2012; Zou et al., 2015). These results demonstrated that *GhCDPK60* was involved in increasing transcription of stress-associated genes in *Arabidopsis*, thereby improving tolerance to drought stress.

### Silencing of *GhCDPK60* decreased drought tolerance of cotton

**Figure 5:**
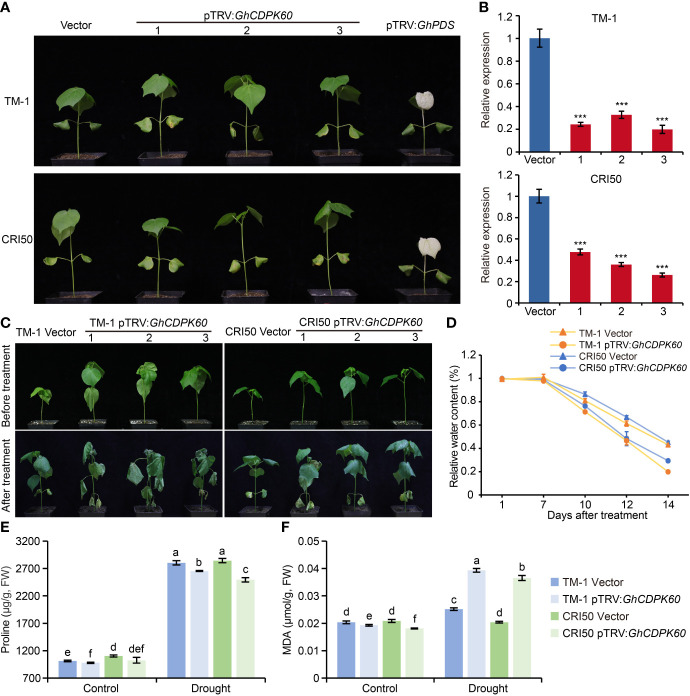
Silencing of *GhCDPK60* decreased tolerance to drought stress in cotton plants. **(A)** Phenotypes of control (lines infected with empty vector) and *GhCDPK60*-silenced plants in two different species of cotton. The *GhPDS* gene was used as an indicator with an albino phenotype on leave after VIGS in cotton. 1-3 indicated three individual plants. **(B)** Relative expression levels of *GhCDPK60* in vector and pTRV : *GhCDPK60* plants. **(C)** Phenotypes of drought-stressed control and *GhCDPK60*-silenced plants in two different species of cotton. Photographs were taken before treatment and two weeks after drought treatment, respectively. **(D)** Relative water content of control and *GhCDPK60*-silenced plants under drought treatment. **(E, F)** The analysis of proline **(E)** and MDA **(F)** content in control and *GhCDPK60*-silenced plants leaves after drought treatment. Values represent the mean ± SE from three biological replicates. ***P< 0.001 by Student’s *t* test. Columns with different letters indicate significant differences (*P*< 0.05, Student’s *t* test).

VIGS is a fast and simple method for transient silencing of genes that is widely used in cotton research (Hayward et al., 2011). To functionally characterize *GhCDPK60* in cotton drought tolerance, *GhCDPK60* was successfully silenced in two different backgrounds using a VIGS strategy (**Figure 5A**). The pTRV : *GhPDS*-inoculated plant leaves showed a bleached phenotype (**Figure 5A**), which was an affirmative indication that the knockdown vector was effective. According to qRT-PCR validation, the expression level of *GhCDPK60* was significantly reduced in the VIGS plants than that in control (plants transformed in empty vector) (**Figure 5B**). To further evaluate the phenotype of the gene-silenced plants under drought stress, the plants were subjected to a two-week drought treatment. We found that the leaves of *GhCDPK60*-silenced plants showed more wilted and shrunken than in control plants, especially in the TM-1 genetic background (**Figure 5C**). The RWC from detached leaves was much lower for the *GhCDPK60*-silenced plants than that in control. In addition, the RWC of *GhCDPK60*-silenced plants in the cv. CRI50 background was slightly higher than that in the TM-1 background (**Figure 5D**), consistent with the dehydration phenotypes. After two-week drought treatment, *GhCDPK60*-silenced plants showed a lower proline content and higher MDA contents, compared with those of vector control (**Figure 5E, F** **)**. To obtain a deeper understanding of the function of *GhCDPK60* under drought stress, we analyzed the transcript levels of some stress-inducible genes and found that they were significant lower in *GhCDPK60*-silenced plants than in control under drought stress (**Supplementary Figure 6**). These results indicated that the silencing of *GhCDPK60* decreased the drought tolerance in cotton.

### *GhCDPK60* contributed to the elimination of ROS

**Figure 6:**
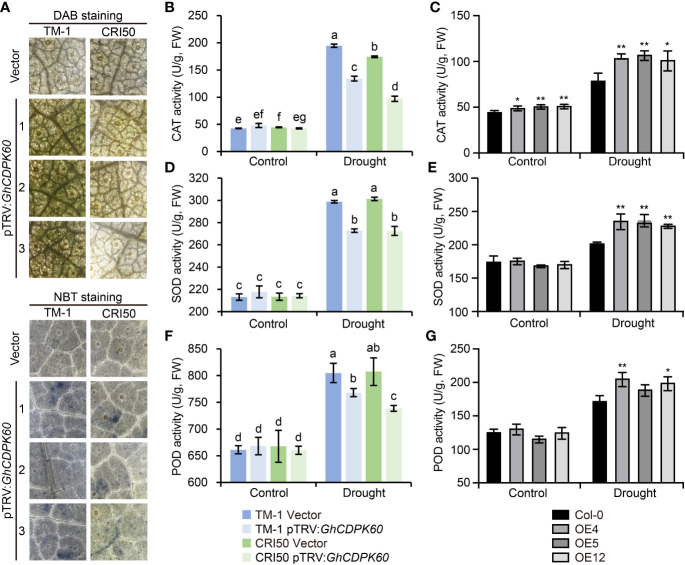
Visualization of ROS and determination of antioxidant enzyme activities. **(A)** H_2_O_2_ and ${\text{O}}_{2}^{-}$ visualization in cotton leaves by staining with DAB and NBT, respectively. **(B-G)** Measurements of CAT **(B, C)**, SOD **(D, E)**, and POD **(F, G)** activities in cotton and *Arabidopsis* leaves after drought treatment, respectively. Statistically significant differences compared to Col-0. Values represent the mean ± SE from three biological replicates. *P< 0.05 and **P< 0.01 by Student’s *t* test. Columns with different letters indicate significant differences (*P*< 0.05, Student’s *t* test).

Increasing evidence suggests that drought induces the production of active oxygen species (Fu and Huang, 2001). ROS production triggers defense mechanisms associated with Ca^2+^ fluxes and ABA signaling under drought stress, but over-accumulation of ROS leads to cell death *via* progressive oxidative damage (Mahmood et al., 2019). Considering the lower MDA content in *GhCDPK60*-OE lines and higher MDA content in *GhCDPK60*-silenced plants under drought stress (**Figure 4F**, **5F**), we hypothesized that GhCDPK60 might be involved in ROS production. In order to determine whether *GhCDPK60* functions on the production of ROS, *GhCDPK60*-silenced plants after drought treatment were stained with 3-3’-diaminobenzidine (DAB) and nitroblue tetrazolium (NBT), respectively, and the results demonstrated that the ROS levels were comparatively higher in *GhCDPK60*-silenced plants in the TM-1 background than that of control (**Figure 6A**), consistent with the phenotypes after drought stress. Plants have developed the scavenging mechanisms to maintain homeostasis of ROS redox reactions and have been protected against the detrimental effects of active oxygen and biotic/abiotic stress by non-enzymatic antioxidants and enzymatic components (Van Breusegem et al., 1998), such as catalase (CAT), superoxide dismutase (SOD), and peroxidase (POD) (Bowler et al., 1992). Subsequently, we determined the CAT, SOD, and POD activities both in transgenic *Arabidopsis* and silenced cotton under drought stress. We found that CAT, SOD, and POD activities in the *GhCDPK60*-overexpressing *Arabidopsis* lines were significantly higher than in the Col, while the *GhCDPK60*-silenced cotton displayed decreased trends compared to the control (**Figure 6B-G**). These results indicated that the *GhCDPK60* was involved in the elimination of ROS in the defense response to drought stress.

## Discussion

### The identification and analysis of calcium-dependent protein kinases in *Gossypium hirsutum*

Plant CDPKs stand for a multigene family of numerous calcium-dependent protein kinases which are vital for several physiological processes, including biotic and abiotic stress tolerance, by participating in signal transduction pathways and inducing downstream effects, such as altered protein phosphorylation and gene expression patterns (Harmon et al., 2001). However, to the best of our knowledge, studies focused on the roles of CDPKs in response to abiotic stress in upland cotton were limited. The release of plant genome sequencing data has enabled the identification of *CDPKs* in *Arabidopsis* (Cheng et al., 2002; Hrabak et al., 2003), *Oryza sativa* (Asano et al., 2005), *Triticum aestivum L.* (Li et al., 2008), *Populus trichocarpa* (Zuo et al., 2013), *Fragaria x ananassa* (Crizel et al., 2020), banana (Li et al., 2020), and *Solanum habrochaites* (Li et al., 2022), which provides models for the characterization of the *CDPKs* gene family in upland cotton. Benefiting from the advent and availability of the published genome sequence in *G. hirsutum* TM-1 (Zhang et al., 2015; Wang et al., 2019; Chen et al., 2020), we identified and analyzed *CDPKs* gene and proteins by extracting and aligning their sequences in TM-1 (HAU_v1) (Wang et al., 2019). Totally, 80 putative *GhCDPKs* were manually reannotated and confirmed in upland cotton, most of which existed as gene pairs in A*t* and D*t* subgenome, while only a few members existed in one of the subgenomes, such as *GhCDPK5*, *GhCDPK6*, and *GhCDPK22*, suggesting that the *GhCDPKs* maybe experience genome replication event. Similar to CDPKs in rice and *Arabidopsis*, all the members of GhCDPKs have typical conserved EF-hand motifs, which contribute to binding Ca^2+^. The phylogenetic analysis on GhCDPKs proteins showed that most of them were greatly conserved during evolution.

### *GhCDPK60* plays a positive role in conferring drought stress tolerance in upland cotton

Drought stress are the major abiotic threats to plants that result in alters in transpiration rate, development and composition of photosynthetic apparatus, excess ROS production, and biochemical composition changes, and further affect plant growth and decrease crop yield. Plants have developed a network of signal transduction pathways to regulate metabolism to adapt to environments, including CDPKs mediated Ca^2+^ pathway (Asano et al., 2012a). Cotton is an essential economic fiber crop with high quality oil and protein seeds, frequently undergoes drought stress, which severely damages the cotton yield. In this research, we found that the *GhCDPK60* was significantly induced by drought and hormones treatments, suggesting its potential function on regulating drought stress tolerance. GhCDPK60 was confirmed to be a plasma membrane associated protein. The plasma membrane localization of GhCDPK60 could facilitate activation of several enzyme by localized calcium signals generated in response to abiotic or biotic stimuli (Kudla et al., 2010). To assess the role of *GhCDPK60* under drought conditions, we employed overexpression assay in *Arabidopsis* and VIGS assay in cotton. Our results demonstrated that *GhCDPK60*-overexpressing plants were stronger than the Col and retained more water content, while *GhCDPK60*-silence plants were more vulnerable to drought stress compared with the control, reflected in the withered leaves and lower leaf relative water content, suggesting that *GhCDPK60* is a positive regulator of the response to drought stress. These results are consistent with those of previous studies on some other *CDPKs* that positively regulate drought stress tolerance (Wei et al., 2014; Zou et al., 2015; Huang et al., 2018). It was noted that phenotypes of *GhCDPK60*-silence plants in the TM-1 background were more severe than that in the CRI50 background under drought stress, suggesting that there existed other genes to help resist to drought stress in CRI50.

To gain a deeper understanding of the molecular mechanism of *GhCDPK60* under drought stress, we analyzed the transcription level of several stress-inducible genes. In previous studies, the *atcat3* mutant displayed a drought stress-sensitive phenotype (Zou et al., 2015). Overexpression of *AtNHX1* gene improved drought tolerance in transgenic groundnut (Asif et al., 2011). The *RD29* (Responsive to Desiccation) genes *RD29A* and *RD29B* were induced by desiccation stress (Jia et al., 2012). AtDREB2A interacts with a cis-acting DRE sequence to activate the expression of downstream genes that are involved in drought stress response in *Arabidopsis* (Sakuma et al., 2006). Under drought stress, the transcription levels of drought-responsive genes, *AtCAT3*, *AtNXH1*, *AtRD29A/B*, and *AtDREB2A* were higher in *GhCDPK60*-overexpressing *Arabidopsis* than in Col. By contrast, our results showed that the transcription levels of drought-related genes *GhCDPK1*, *GhZEP*, *GhAAO3*, and *GhABA2* were lower in *GhCDPK60*-silence cotton than control. *GhCDPK1*, as a positive regulator, was induced by drought stress (Tian et al., 2016). *AtZEP*, a homologous gene of *GhZEP*, played important roles in response to osmotic stress (Park et al., 2008). Two ABA biosynthetic-related genes *GhAAO3* and *GhABA2* were homologous with *AtAAO3* and *AtABA2*, respectively, which were required for drought tolerance (Khan et al., 2019). Considering the result that *GhCDPK60* responded to ABA signal, we speculated that *GhCDPK60* may play role in increasing expression of stress-associated genes and ABA-regulated drought stress tolerance.

### *GhCDPK60* increases drought tolerance by accumulating more proline and reducing the accumulation of ROS

The ability to reserve enough water is crucial for plants to overcome drought stress. Our results showed that *GhCDPK60* played a positive role in improving the ability of the plant to retain water under dehydration conditions. Subsequently, we explored the physiological mechanism by which *GhCDPK60* enables the plants to retain water. Plants are inclined to accumulate compatible osmolytes such as proline to reduce the cellular osmotic potential under limited water conditions (Per et al., 2017). Our results showed that there were increased proline content in *GhCDPK60*-overexpressing *Arabidopsis*, but decreased content in *GhCDPK60*-silence cotton. Hence, we deduced that *GhCDPK60* functions in osmotic adjustment and improvement of ability to retain water during drought in plants. Generally, plants tend to generate ROS under abiotic or biotic stress conditions, thereby impairing the production of biomolecules and increasing the MDA concentration as well as the permeability of the plasma membrane. MDA is produced by polyunsaturated fatty acid peroxidation in the cells, which is a robust diagnostic indicator for determining the injury degree to a stressed plant (Morales and Munné-Bosch, 2019). In our research, *GhCDPK60*-overexpressing *Arabidopsis* produced less MDA than Col under drought stress, whereas *GhCDPK60*-silence cotton produced more compared with control, suggesting that expression of *GhCDPK60* might alleviate the damages induced by oxidative stress. Plants have developed a complicated ROS scavenging system to minimize and/or prevent oxidative damage in cells (Van Breusegem et al., 1998). Indeed, *GhCDPK60*-silence cotton exhibited obvious reduced activities of antioxidant enzymes under drought stress, such as CAT, SOD, and POD, on the contrary, *GhCDPK60*-overexpressing *Arabidopsis* showed enhanced enzymes activities. These results are consistent with that *GhCDPK60*-overexpressing *Arabidopsis* displayed increased drought stress tolerance, which possibly as a result of reduced ROS accumulation. Overall, *GhCDPK60* could enhance the ability of plants to resist drought stress by accumulating more osmotic adjustment substances, and enhancing the activity of the antioxidant system to scavenge ROS.

## Conclusion

In this study, a total of 80 upland cotton *CDPK* genes were identified. Their conserved motifs shared a notable similarity with *Arabidopsis* and rice, which leads to conserved functions. We found that *GhCDPK60* was significantly induced in drought and hormone treatments. The overexpression of *GhCDPK60* enhanced the ability of plants to resist to osmotic stress, while silencing of *GhCDPK60* severely compromised the drought tolerance of upland cotton. The results showed that *GhCDPK60* could augment drought stress tolerance *via* the induction of expression of stress-related genes expression, osmotic regulation, and ROS scavenging. Our study revealed that *GhCDPK60* positively regulated the drought stress tolerance of upland cotton, providing a new gene resource for the genetic improvement of drought tolerance in upland cotton. Future studies should investigate the signaling networks and biochemical functions of *GhCDPK60* to gain a deeper understanding of its molecular mechanisms for regulating drought stress tolerance.

## Data availability statement

Publicly available datasets were analyzed in this study. This data can be found here: https://www.cottongen.org.

## Author contributions

SY and ZFeng designed the project and revised the manuscript. MY, XY, GZ, DS, and CH performed the experiments. MY and XY performed data analysis and wrote the manuscript. Other authors provided technical supports. All authors contributed to the article and approved the submitted version.

## Funding

This research was sponsored by the Program for Research and Development of Zhejiang A&F University (2022LFR108).

## Acknowledgments

Thanks for College of Advanced Agricultural Sciences, Zhejiang A&F University.

## Conflict of interest

The authors declare that the research was conducted in the absence of any commercial or financial relationships that could be construed as a potential conflict of interest.

## Publisher’s note

All claims expressed in this article are solely those of the authors and do not necessarily represent those of their affiliated organizations, or those of the publisher, the editors and the reviewers. Any product that may be evaluated in this article, or claim that may be made by its manufacturer, is not guaranteed or endorsed by the publisher.

## Supplementary material

Supplementary Figure 1 Chromosomal distribution of *GhCDPKs*.

Supplementary Figure 2 Distribution of conserved motifs in GhCDPK proteins. Colored boxes indicate putative motifs. aa, amino acid.

Supplementary Figure 3 Expression heatmap of *GhCDPK* genes in different tissues and organs. The colors varied from yellow to blue represent the scales of the relative expression levels. The fragments per kilobase of transcript per million mapped reads (FPKM) values of *GhCDPK* genes in different tissues and organs were from public RNA-seq data. DPA, day post-anthesis.

Supplementary Figure 4 Subcellular localization of the GhCDPK60-GFP fusion protein in *N. benthamiana* leaf cells. Empty GFP protein was used as positive control. Bars = 20 μm.

Supplementary Figure 5 Relative expression of five stress-related genes, including *AtCAT3* (A), *AtNXH1* (B), *AtRD29A/B* (C-D), and *AtDREB2A* (E) in Col-0 and *GhCDPK60*-overexpressing plants after drought treatment. Values represent the mean ± SE from three biological replicates. **P*< 0.05 and ****P<* 0.001 by Student’s *t* test.

Supplementary Figure 6 Relative expression of four stress-related genes, including *GhCDPK1* (A), *GhZEP* (B), *GhAAO3* (C), and *GhABA2* (D) in control and *GhCDPK60*-silenced plants after drought treatment. V, lines infected with empty vector. 1-3 indicated three individual plants. Statistically significant differences compared to control (lines infected with empty vector). Values represent the mean ± SE from three biological replicates. **P*< 0.05, ***P*< 0.01 and ****P<* 0.001 by Student’s *t* test.

## References

[B1] Alici E. H. Arabaci G. (2016). Determination of SOD, POD, PPO and cat enzyme activities in *Rumex obtusifolius* l. Annu. Res. Rev. Biol. 11 (3), 1–7. doi: 10.9734/ARRB/2016/29809

[B2] Artico S. Nardeli S. M. Brilhante O. Grossi-de-Sa M. F. Alves-Ferreira M. (2010). Identification and evaluation of new reference genes in gossypium hirsutum for accurate normalization of real-time quantitative RT-PCR data. BMC Plant Biol. 10, 49. doi: 10.1186/1471-2229-10-49 20302670 PMC2923523

[B3] Asano T. Hayashi N. Kikuchi S. Ohsugi R. (2012a). CDPK-mediated abiotic stress signaling. Plant Signal Behav. 7, 817–821. doi: 10.4161/psb.20351 22751324 PMC3583972

[B4] Asano T. Hayashi N. Kobayashi M. Aoki N. Miyao A. Mitsuhara I. . (2012b). A rice calcium-dependent protein kinase OsCPK12 oppositely modulates salt-stress tolerance and blast disease resistance. Plant J. 69, 26–36. doi: 10.1111/j.1365-313X.2011.04766.x 21883553

[B5] Asano T. Tanaka N. Yang G. Hayashi N. Komatsu S. (2005). Genome-wide identification of the rice calcium-dependent protein kinase and its closely related kinase gene families: Comprehensive analysis of the *CDPKs* gene family in rice. Plant Cell Physiol. 46, 356–366. doi: 10.1093/pcp/pci035 15695435

[B6] Asif M. A. Zafar Y. Iqbal J. Iqbal M. M. Rashid U. Ali G. M. . (2011). Enhanced expression of *AtNHX1*, in transgenic groundnut (*Arachis hypogaea* l.) improves salt and drought tolerence. Mol. Biotechnol. 49, 250–256. doi: 10.1007/s12033-011-9399-1 21455815

[B7] Bowler C. Montagu M. V. Inze D. (1992). Superoxide dismutase and stress tolerance. Annu. Rev. Plant Physiol. Plant Mol. Biol. 43, 83–116. doi: 10.1146/annurev.pp.43.060192.000503

[B8] Cheng S. H. Willmann M. R. Chen H. C. Sheen J. (2002). Calcium signaling through protein kinases. the arabidopsis calcium-dependent protein kinase gene family. Plant Physiol. 129, 469–485. doi: 10.1104/pp.005645 12068094 PMC1540234

[B9] Chen Z. J. Sreedasyam A. Ando A. Song Q. De Santiago L. M. Hulse-Kemp A. M. . (2020). Genomic diversifications of five *Gossypium* allopolyploid species and their impact on cotton improvement. Nat. Genet. 52, 525–533. doi: 10.1038/s41588-020-0614-5 32313247 PMC7203012

[B10] Chen Y. Zhou X. Chang S. Chu Z. Wang H. Han S. . (2017). Calcium-dependent protein kinase 21 phosphorylates 14-3-3 proteins in response to ABA signaling and salt stress in rice. Biochem. Biophys. Res. Commun. 493, 1450–1456. doi: 10.1016/j.bbrc.2017.09.166 28988107

[B11] Clough S. J. Bent A. F. (1998). Floral dip: A simplified method for *Agrobacterium*-mediated transformation of arabidopsis thaliana. Plant J. 16, 735–743. doi: 10.1046/j.1365-313x.1998.00343.x 10069079

[B12] Coca M. San Segundo B. (2010). AtCPK1 calcium-dependent protein kinase mediates pathogen resistance in arabidopsis. Plant J. 63, 526–540. doi: 10.1111/j.1365-313X.2010.04255.x 20497373

[B13] Crizel R. L. Perin E. C. Vighi I. L. Woloski R. Seixas A. Da Silva Pinto L. . (2020). Genome-wide identification, and characterization of the *CDPK* gene family reveal their involvement in abiotic stress response in fragaria x ananassa. Sci. Rep. 10, 11040. doi: 10.1038/s41598-020-67957-9 32632235 PMC7338424

[B14] Cutler S. R. Rodriguez P. L. Finkelstein R. R. Abrams S. R. (2010). Abscisic acid: Emergence of a core signaling network. Annu. Rev. Plant Biol. 61, 651–679. doi: 10.1146/annurev-arplant-042809-112122 20192755

[B15] Fang L. Ma L. Zhao S. Cao R. Jiao G. Hu P. . (2022). Alanine aminotransferase (OsAlaAT1) modulates nitrogen utilization, grain yield, and quality in rice. J. Genet. Genomics 49, 510–513. doi: 10.1016/j.jgg.2022.02.028 35341969

[B16] Fang Y. Xiong L. (2015). General mechanisms of drought response and their application in drought resistance improvement in plants. Cell Mol. Life Sci. 72, 673–689. doi: 10.1007/s00018-014-1767-0 25336153 PMC11113132

[B17] Finn R. D. Clements J. Eddy S. R. (2011). HMMER web server: Interactive sequence similarity searching. Nucleic Acids Res. 39, W29–W37. doi: 10.1093/nar/gkr367 21593126 PMC3125773

[B18] Fu J. Huang B. (2001). Involvement of antioxidants and lipid peroxidation in the adaptation of two cool-season grasses to localized drought stress. Environ. Exp. Bot. 45, 105–114. doi: 10.1016/S0098-8472(00)00084-8 11275219

[B19] Group M. Ichimura K. Shinozaki K. Tena G. Sheen J. Henry Y. . (2002). Mitogen-activated protein kinase cascades in plants: A new nomenclature. Trends Plant Sci. 7, 301–308. doi: 10.1016/S1360-1385(02)02302-6 12119167

[B20] Gu Z. Huang C. Li F. Zhou X. (2014). A versatile system for functional analysis of genes and microRNAs in cotton. Plant Biotechnol. J. 12, 638–649. doi: 10.1111/pbi.12169 24521483

[B21] Harmon A. C. Gribskov M. Gubrium E. Harper J. F. (2001). The CDPK superfamily of protein kinases. New Phytol. 151, 175–183. doi: 10.1046/j.1469-8137.2001.00171.x 33873379

[B22] Hayward A. Padmanabhan M. Dinesh-Kumar S. P. (2011). Virus-induced gene silencing in *Nicotiana benthamiana* and other plant species. Methods Mol. Biol. 678, 55–63. doi: 10.1007/978-1-60761-682-5_5 20931372

[B23] Hrabak E. M. Chan C. W. Gribskov M. Harper J. F. Choi J. H. Halford N. . (2003). The arabidopsis CDPK-SnRK superfamily of protein kinases. Plant Physiol. 132, 666–680. doi: 10.1104/pp.102.011999 12805596 PMC167006

[B24] Huang K. Peng L. Liu Y. Yao R. Liu Z. Li X. . (2018). Arabidopsis calcium-dependent protein kinase AtCPK1 plays a positive role in salt/drought-stress response. Biochem. Biophys. Res. Commun. 498, 92–98. doi: 10.1016/j.bbrc.2017.11.175 29196259

[B25] Hu W. Huang C. Deng X. Zhou S. Chen L. Li Y. . (2013). *TaASR1*, a transcription factor gene in wheat, confers drought stress tolerance in transgenic tobacco. Plant Cell Environ. 36, 1449–1464. doi: 10.1111/pce.12074 23356734

[B26] Irigoyen J. Einerich D. Sánchez-Díaz M. (1992). Water stress induced changes in concentrations of proline and total soluble sugars in nodulated alfalfa (Medicago sativd) plants. Physiol. Plant. 84, 55–60. doi: 10.1111/j.1399-3054.1992.tb08764.x

[B27] Jambunathan N. (2010). Determination and detection of reactive oxygen species (ROS), lipid peroxidation, and electrolyte leakage in plants. Methods Mol. Biol. 639, 292–298. doi: 10.1007/978-1-60761-702-0_18 20387054

[B28] Jiang S. Zhang D. Wang L. Pan J. Liu Y. Kong X. . (2013). A maize calcium-dependent protein kinase gene, *ZmCPK4*, positively regulated abscisic acid signaling and enhanced drought stress tolerance in transgenic arabidopsis. Plant Physiol. Biochem. 71, 112–120. doi: 10.1016/j.plaphy.2013.07.004 23911729

[B29] Jia H. Zhang S. Ruan M. Wang Y. Wang C. (2012). Analysis and application of *RD29* genes in abiotic stress response. Acta Physiol. Plant. 34, 1239–1250. doi: 10.1007/s11738-012-0969-z

[B30] Jogawat A. Yadav B. Chhaya Lakra N. Singh A. K. Narayan O. P. (2021). Crosstalk between phytohormones and secondary metabolites in the drought stress tolerance of crop plants: A review. Physiol. Plant 172, 1106–1132. doi: 10.1111/ppl.13328 33421146

[B31] Khan M. Imran Q. M. Shahid M. Mun B.-G. Lee S.-U. Khan M. A. . (2019). Nitric oxide- induced AtAO3 differentially regulates plant defense and drought tolerance in arabidopsis thaliana. BMC Plant Biol. 19, 602. doi: 10.1186/s12870-019-2210-3 31888479 PMC6937950

[B32] Klimecka M. Muszyńska G. (2007). Structure and functions of plant calcium-dependent protein kinases. Acta Biochim. Pol. 54, 219–233. doi: 10.18388/abp.2007_3242 17446936

[B33] Kolukisaoglu U. Weinl S. Blazevic D. Batistic O. Kudla J. (2004). Calcium sensors and their interacting protein kinases: Genomics of the arabidopsis and rice CBL-CIPK signaling networks. Plant Physiol. 134, 43–58. doi: 10.1104/pp.103.033068 14730064 PMC316286

[B34] Kudla J. Batistic O. Hashimoto K. (2010). Calcium signals: The lead currency of plant information processing. Plant Cell 22, 541–563. doi: 10.1105/tpc.109.072686 20354197 PMC2861448

[B35] Kumar S. Stecher G. Tamura K. (2016). MEGA7: Molecular evolutionary genetics analysis version 7.0 for bigger datasets. Mol. Biol. Evol. 33, 1870–1874. doi: 10.1093/molbev/msw054 27004904 PMC8210823

[B36] Liese A. Romeis T. (2013). Biochemical regulation of *in vivo* function of plant calcium-dependent protein kinases (CDPK). Biochim. Biophys. Acta 1833, 1582–1589. doi: 10.1016/j.bbamcr.2012.10.024 23123193

[B37] Li F. Fan G. Lu C. Xiao G. Zou C. Kohel R. J. . (2015a). Genome sequence of cultivated upland cotton (Gossypium hirsutum TM-1) provides insights into genome evolution. Nat. Biotechnol. 33, 524–530. doi: 10.1038/nbt.3208 25893780

[B38] Li M. Hu W. Ren L. Jia C. Liu J. Miao H. . (2020). Identification, expression, and interaction network analyses of the *CDPK* gene family reveal their involvement in the development, ripening, and abiotic stress response in banana. Biochem. Genet. 58, 40–62. doi: 10.1007/s10528-019-09916-2 31144068

[B39] Li H. M. Liu S. D. Ge C. W. Zhang X. M. Zhang S. P. Chen J. . (2019). Analysis of drought tolerance and associated traits in upland cotton at the seedling stage. Int. J. Mol. Sci. 20, 3888. doi: 10.3390/ijms20163888 31404956 PMC6720584

[B40] Li L. B. Yu D. W. Zhao F. L. Pang C. Y. Song M. Z. Wei H. L. . (2015b). Genome-wide analysis of the calcium-dependent protein kinase gene family in gossypium raimondii. J. Integr. Agric. 14, 29–41. doi: 10.1016/S2095-3119(14)60780-2

[B41] Li L. B. Zhang C. Huang J. Q. Liu Q. B. Wei H. L. Wang H. T. . (2021). Genomic analyses reveal the genetic basis of early maturity and identification of loci and candidate genes in upland cotton (*Gossypium hirsutum* l.). Plant Biotechnol. J. 1, 109–123. doi: 10.1111/pbi.13446 PMC7769233 32652678

[B42] Li Y. Zhang H. Liang S. Chen X. Liu J. Zhang Y. . (2022). Identification of *CDPK* gene family in solanum habrochaites and its function analysis under stress. Int. J. Mol. Sci. 23, 4227. doi: 10.3390/ijms23084227 35457042 PMC9031491

[B43] Li A. L. Zhu Y. F. Tan X. M. Wang X. Wei B. Guo H. Z. . (2008). Evolutionary and functional study of the *CDPK* gene family in wheat (Triticum aestivum l.). Plant Mol. Biol. 66, 429–443. doi: 10.1007/s11103-007-9281-5 18185910

[B44] Liu N. Hake K. Wang W. Zhao T. Romeis T. Tang D. (2017). CALCIUM-DEPENDENT PROTEIN KINASE5 associates with the truncated NLR protein TIR-NBS2 to contribute to exo70B1-mediated immunity. Plant Cell 29, 746–759. doi: 10.1105/tpc.16.00822 28351987 PMC5435426

[B45] Liu W. Li W. He Q. Daud M. K. Chen J. Zhu S. (2014). Genome-wide survey and expression analysis of calcium-dependent protein kinase in gossypium raimondii. PloS One 9, e98189. doi: 10.1371/journal.pone.0098189 24887436 PMC4041719

[B46] Livak K. J. Schmittgen T. D. (2001). Analysis of relative gene expression data using real-time quantitative PCR and the 2(-delta delta C(T)) method. Methods 25, 402–408. doi: 10.1006/meth.2001.1262 11846609

[B47] Mahmood T. Khalid S. Abdullah M. Ahmed Z. Shah M. K. N. Ghafoor A. . (2019). Insights into drought stress signaling in plants and the molecular genetic basis of cotton drought tolerance. Cells 9, 105. doi: 10.3390/cells9010105 31906215 PMC7016789

[B48] Ma P. Liu J. Yang X. Ma R. (2013). Genome-wide identification of the maize calcium-dependent protein kinase gene family. Appl. Biochem. Biotechnol. 169, 2111–2125. doi: 10.1007/s12010-013-0125-2 23397323

[B49] Ma S. Y. Wu W. H. (2007). AtCPK23 functions in arabidopsis responses to drought and salt stresses. Plant Mol. Biol. 65, 511–518. doi: 10.1007/s11103-007-9187-2 17541706

[B50] McCormack E. Tsai Y. C. Braam J. (2005). Handling calcium signaling: Arabidopsis CaMs and CMLs. Trends Plant Sci. 10, 383–389. doi: 10.1016/j.tplants.2005.07.001 16023399

[B51] Morales M. Munné-Bosch S. (2019). Malondialdehyde: Facts and artifacts. Plant Physiol. 180, 1246–1250. doi: 10.1104/pp.19.00405 31253746 PMC6752910

[B52] Park H. Y. Seok H. Y. Park B. K. Kim S. H. Goh C. H. Lee B. H. . (2008). Overexpression of arabidopsis ZEP enhances tolerance to osmotic stress. Biochem. Biophys. Res. Commun. 375, 80–85. doi: 10.1016/j.bbrc.2008.07.128 18680727

[B53] Per T. S. Khan N. A. Reddy P. S. Masood A. Hasanuzzaman M. Khan M. I. R. . (2017). Approaches in modulating proline metabolism in plants for salt and drought stress tolerance: Phytohormones, mineral nutrients and transgenics. Plant Physiol. Biochem. 115, 126–140. doi: 10.1016/j.plaphy.2017.03.018 28364709

[B54] Ray S. Agarwal P. Arora R. Kapoor S. Tyagi A. K. (2007). Expression analysis of calcium-dependent protein kinase gene family during reproductive development and abiotic stress conditions in rice (Oryza sativa l. ssp. indica). Mol. Genet. Genomics 278, 493–505. doi: 10.1007/s00438-007-0267-4 17636330

[B55] Reyes M. I. Flores-Vergara M. A. Guerra-Peraza O. Rajabu C. Desai J. Hiromoto-Ruiz Y. H. . (2017). A VIGS screen identifies immunity in the arabidopsis pla-1 accession to viruses in two different genera of the geminiviridae. Plant J. 92, 796–807. doi: 10.1111/tpj.13716 28901681 PMC5725698

[B56] Sakuma Y. Maruyama K. Qin F. Osakabe Y. Shinozaki K. Yamaguchi-Shinozaki K. (2006). Dual function of an arabidopsis transcription factor DREB2A in water-stress-responsive and heat-stress-responsive gene expression. Proc. Natl. Acad. Sci. U.S.A. 103, 18822–18827. doi: 10.1073/pnas.0605639103 17030801 PMC1693746

[B57] Saranga Y. Paterson A. H. Levi A. (2009). Bridging classical and molecular genetics of abiotic stress resistance in Cotton. Genet. Genom. Cott. 3, 337–52.

[B58] Schmedes A. Hølmer G. (1989). A new thiobarbituric acid (TBA) method for determining free malondialdehyde (MDA) and hydroperoxides selectively as a measure of lipid peroxidation. J. Am. Oil Chemists’ Soc. 66, 813–817. doi: 10.1007/BF02653674

[B59] Sun Z. Li S. Chen W. Zhang J. Zhang L. Sun W. . (2021). Plant dehydrins: Expression, regulatory networks, and protective roles in plants challenged by abiotic stress. Int. J. Mol. Sci. 23, 12619. doi: 10.3390/ijms222312619 PMC8657568 34884426

[B60] Tian X. H. Zhang M. D. Pang X. B. Zhu J. B. Zhu X. X. (2016). Preliminary exploration for function of cotton GhCDPK1 gene under drought stress. Acta Botanica Boreali-Occidentalia Sin. 36, 1515–1521.

[B61] Ullah A. Sun H. Hakim Yang X. Zhang X. (2018). A novel cotton WRKY gene, GhWRKY6-like, improves salt tolerance by activating the ABA signaling pathway and scavenging of reactive oxygen species. Physiol. Plant 162, 439–454. doi: 10.1111/ppl.12651 29027659

[B62] Valmonte G. R. Arthur K. Higgins C. M. Macdiarmid R. M. (2014). Calcium-dependent protein kinases in plants: evolution, expression and function. Plant Cell Physiol. 55, 551–569. doi: 10.1093/pcp/pct200 24363288

[B63] Van Breusegem F. Van Montagu M. Inzé D. (1998). Engineering stress tolerance in maize. Outlook Agric. 27, 115–124. doi: 10.1177/003072709802700209

[B64] Wang N. Liu Y. Cong Y. Wang T. Zhong X. Yang S. . (2016). Genome-wide identification of soybean U-box E3 ubiquitin ligases and roles of GmPUB8 in negative regulation of drought stress response in arabidopsis. Plant Cell Physiol. 57, 1189–1209. doi: 10.1093/pcp/pcw068 27057003

[B65] Wang H. Mei W. Qin Y. Zhu Y. (2011). 1-Aminocyclopropane-1-carboxylic acid synthase 2 is phosphorylated by calcium-dependent protein kinase 1 during cotton fiber elongation. Acta Biochim. Biophys. Sin. (Shanghai) 43, 654–661. doi: 10.1093/abbs/gmr056 21742672

[B66] Wang M. Tu L. Yuan D. Zhu D. Shen C. Li J. . (2019). Reference genome sequences of two cultivated allotetraploid cottons, gossypium hirsutum and gossypium barbadense. Nat. Genet. 51, 224–229. doi: 10.1038/s41588-018-0282-x 30510239

[B67] Weckwerth P. Ehlert B. Romeis T. (2015). ZmCPK1, a calcium-independent kinase member of the zea mays CDPK gene family, functions as a negative regulator in cold stress signalling. Plant Cell Environ. 38, 544–558. doi: 10.1111/pce.12414 25052912

[B68] Wei S. Hu W. Deng X. Zhang Y. Liu X. Zhao X. . (2014). A rice calcium-dependent protein kinase OsCPK9 positively regulates drought stress tolerance and spikelet fertility. BMC Plant Biol. 14, 133. doi: 10.1186/1471-2229-14-133 24884869 PMC4036088

[B69] Wernimont A. K. Artz J. D. Finerty P. Jr. Lin Y. H. Amani M. Allali-Hassani A. . (2010). Structures of apicomplexan calcium-dependent protein kinases reveal mechanism of activation by calcium. Nat. Struct. Mol. Biol. 17, 596–601. doi: 10.1038/nsmb.1795 20436473 PMC3675764

[B70] Xia L. Mar Marquès-Bueno M. Bruce C. G. Karnik R. (2019). Unusual roles of secretory SNARE SYP132 in plasma membrane h(+)-ATPase traffic and vegetative plant growth. Plant Physiol. 180, 837–858. doi: 10.1104/pp.19.00266 30926657 PMC6548232

[B71] Yang J. Kim S.-R. Lee S.-K. Choi H. Jeon J.-S. An G. (2015). Alanine aminotransferase 1 (OsAlaAT1) plays an essential role in the regulation of starch storage in rice endosperm. Plant Sci. 240, 79–89. doi: 10.1016/j.plantsci.2015.07.027 26475189

[B72] Zhang T. Hu Y. Jiang W. Fang L. Guan X. Chen J. . (2015). Sequencing of allotetraploid cotton (Gossypium hirsutum l. acc. TM-1) provides a resource for fiber improvement. Nat. Biotechnol. 33, 531–537. doi: 10.1038/nbt.3207 25893781

[B73] Zhang Y. Luan Q. Jiang J. Li Y. (2021a). Prediction and utilization of malondialdehyde in exotic pine under drought stress using near-infrared spectroscopy. Front. Plant Sci. 12, 735275. doi: 10.3389/fpls.2021.735275 34733301 PMC8558207

[B74] Zhang X. Ren Z. Hu G. Wei H. Fan S. Ma Q. (2021b). Functional divergence of AP1 and FUL genes related to flowering regulation in upland cotton (*Gossypium hirsutum* l.). Research Square. doi: 10.21203/rs.3.rs-817648/v1 35777126

[B75] Zhao R. Sun H. Zhao N. Jing X. Shen X. Chen S. (2015). The arabidopsis Ca²^+^-dependent protein kinase CPK27 is required for plant response to salt-stress. Gene 563, 203–214. doi: 10.1016/j.gene.2015.03.024 25791495

[B76] Zhu J. K. (2002). Salt and drought stress signal transduction in plants. Annu. Rev. Plant Biol. 53, 247–273. doi: 10.1146/annurev.arplant.53.091401.143329 12221975 PMC3128348

[B77] Zhu Y. N. Shi D. Q. Ruan M. B. Zhang L. L. Meng Z. H. Liu J. . (2013). Transcriptome analysis reveals crosstalk of responsive genes to multiple abiotic stresses in cotton (*Gossypium hirsutum* l.). PloS One 11, e80218. doi: 10.1371/journal.pone.0080218 PMC3818253 24224045

[B78] Zhu S. Y. Yu X. C. Wang X. J. Zhao R. Li Y. Fan R. C. . (2007). Two calcium-dependent protein kinases, CPK4 and CPK11, regulate abscisic acid signal transduction in arabidopsis. Plant Cell 19, 3019–3036. doi: 10.1105/tpc.107.050666 17921317 PMC2174700

[B79] Zou J. J. Li X. D. Ratnasekera D. Wang C. Liu W. X. Song L. F. . (2015). Arabidopsis CALCIUM-DEPENDENT PROTEIN KINASE8 and CATALASE3 function in abscisic acid-mediated signaling and H2O2 homeostasis in stomatal guard cells under drought stress. Plant Cell 27, 1445–1460. doi: 10.1105/tpc.15.00144 25966761 PMC4456645

[B80] Zou J. J. Wei F. J. Wang C. Wu J. J. Ratnasekera D. Liu W. X. . (2010). Arabidopsis calcium-dependent protein kinase CPK10 functions in abscisic acid- and Ca2+-mediated stomatal regulation in response to drought stress. Plant Physiol. 154, 1232–1243. doi: 10.1104/pp.110.157545 20805328 PMC2971602

[B81] Zuo R. Hu R. Chai G. Xu M. Qi G. Kong Y. . (2013). Genome-wide identification, classification, and expression analysis of CDPK and its closely related gene families in poplar (Populus trichocarpa). Mol. Biol. Rep. 40, 2645–2662. doi: 10.1007/s11033-012-2351-z 23242656

