# Supplementary figures and images for: GhCDPK60 positively regulates drought stress tolerance in both transgenic Arabidopsis and cotton by regulating proline content and ROS level
Source: Front Plant Sci. 2022 Dec 1;13:1072584. doi: 10.3389/fpls.2022.1072584 (PMC9751749; doi:10.3389/fpls.2022.1072584)

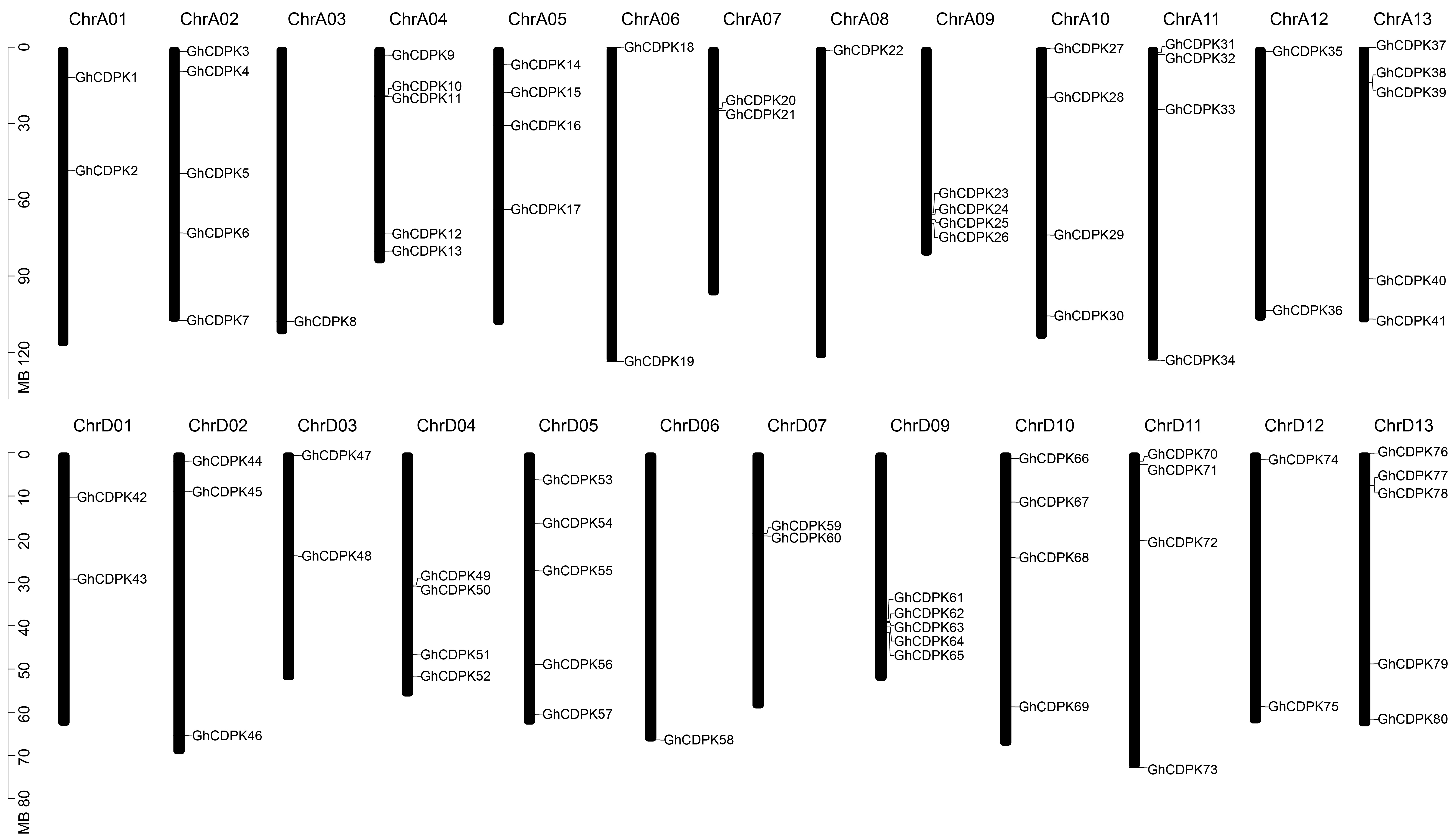

Supplement: Supplementary Figure 1 — Chromosomal distribution of GhCDPKs. [file Image_1.jpg]

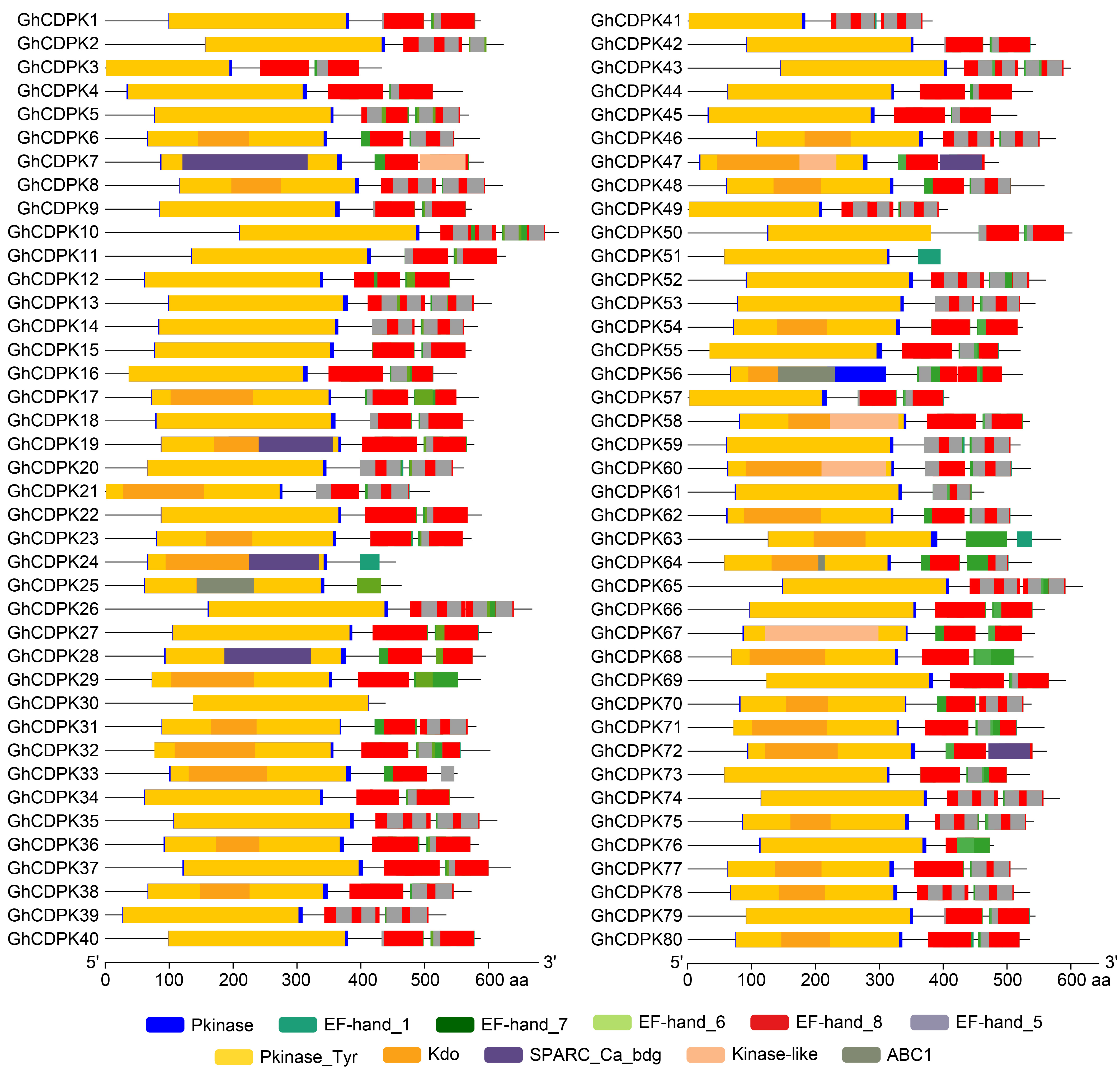

Supplement: Supplementary Figure 2 — Distribution of conserved motifs in GhCDPK proteins. Colored boxes indicate putative motifs. aa, amino acid. [file Image_2.jpg]

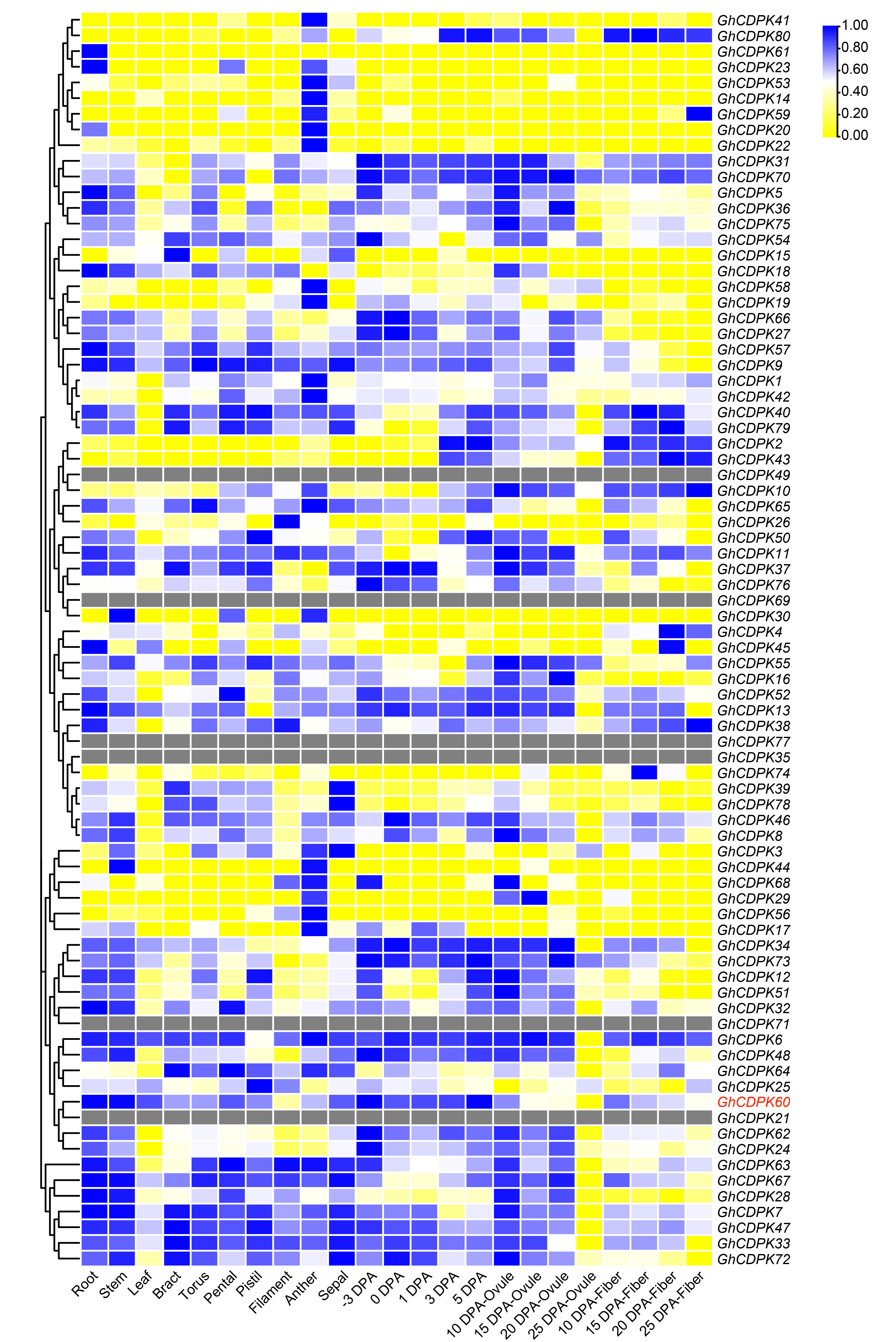

Supplement: Supplementary Figure 3 — Expression heatmap of GhCDPK genes in different tissues and organs. The colors varied from yellow to blue represent the scales of the relative expression levels. The fragments per kilobase of transcript per million mapped reads (FPKM) values of GhCDPK genes in different tissues and organs were from public RNA-seq data. DPA, day post-anthesis. [file Image_3.jpg]

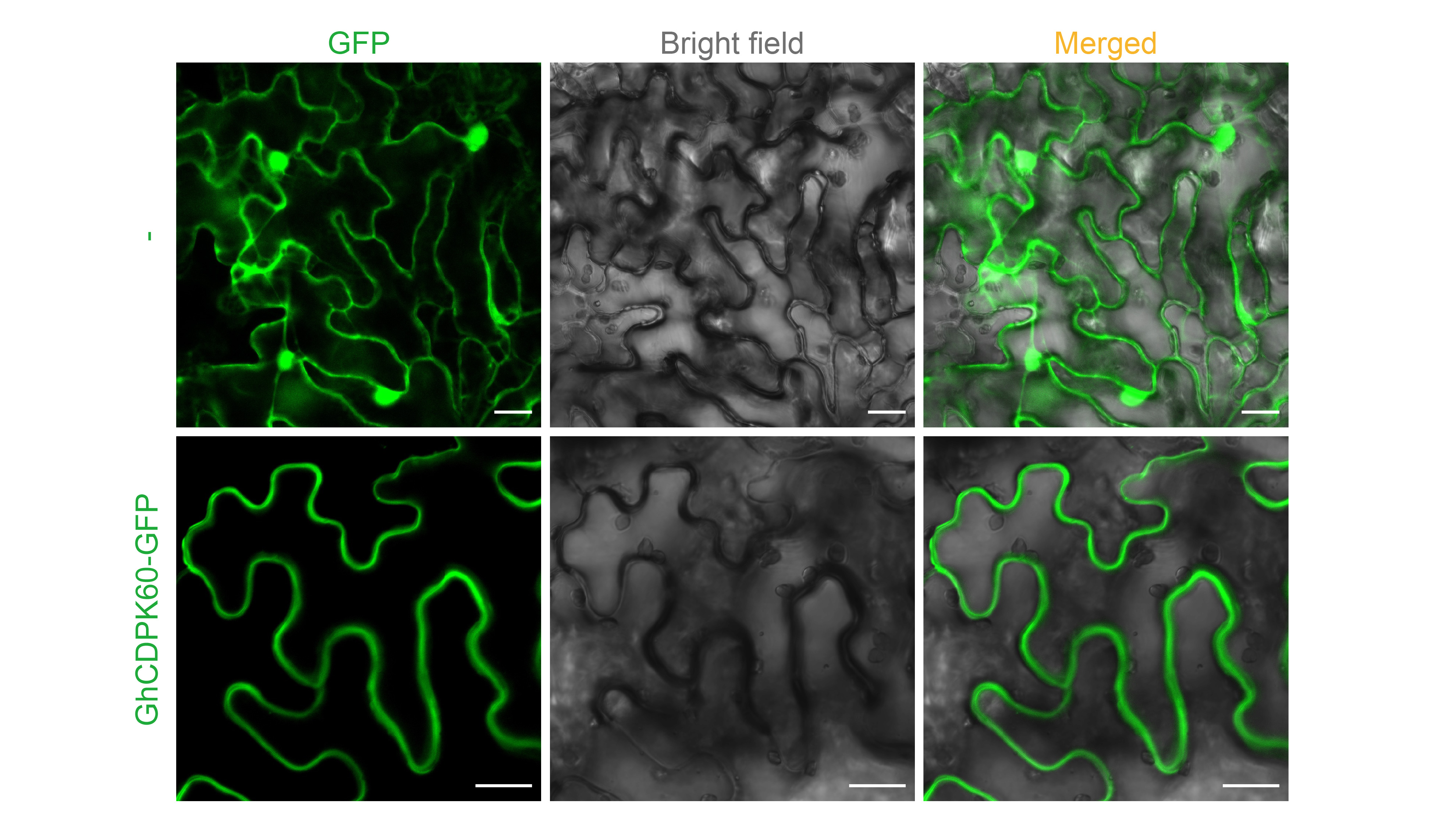

Supplement: Supplementary Figure 4 — Subcellular localization of the GhCDPK60-GFP fusion protein in N. benthamiana leaf cells. Empty GFP protein was used as positive control. Bars = 20 μm. [file Image_4.jpg]

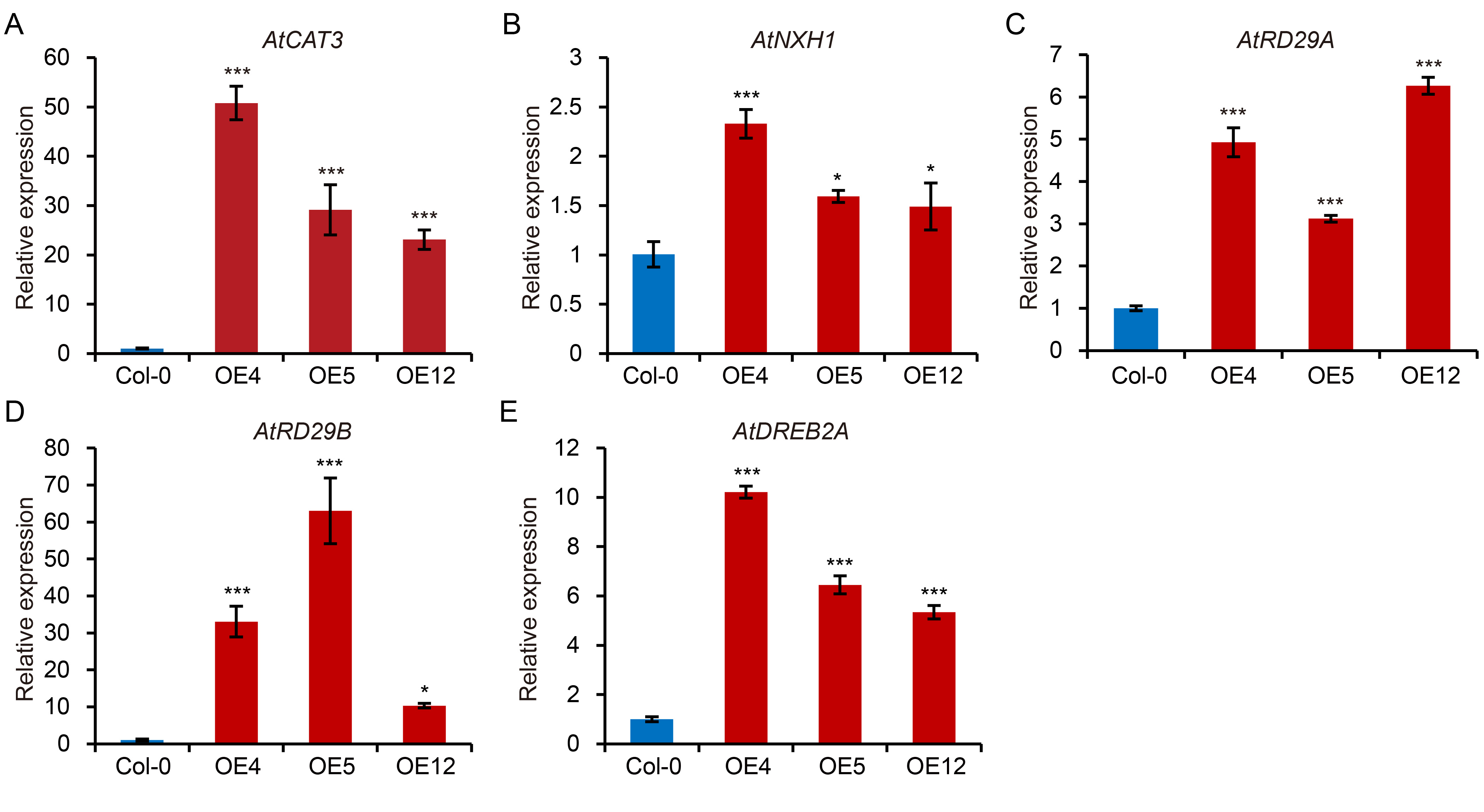

Supplement: Supplementary Figure 5 — Relative expression of five stress-related genes, including AtCAT3 (A), AtNXH1 (B), AtRD29A/B (C-D), and AtDREB2A (E) in Col-0 and GhCDPK60-overexpressing plants after drought treatment. Values represent the mean ± SE from three biological replicates. *P< 0.05 and ***P< 0.001 by Student’s t test. [file Image_5.jpg]

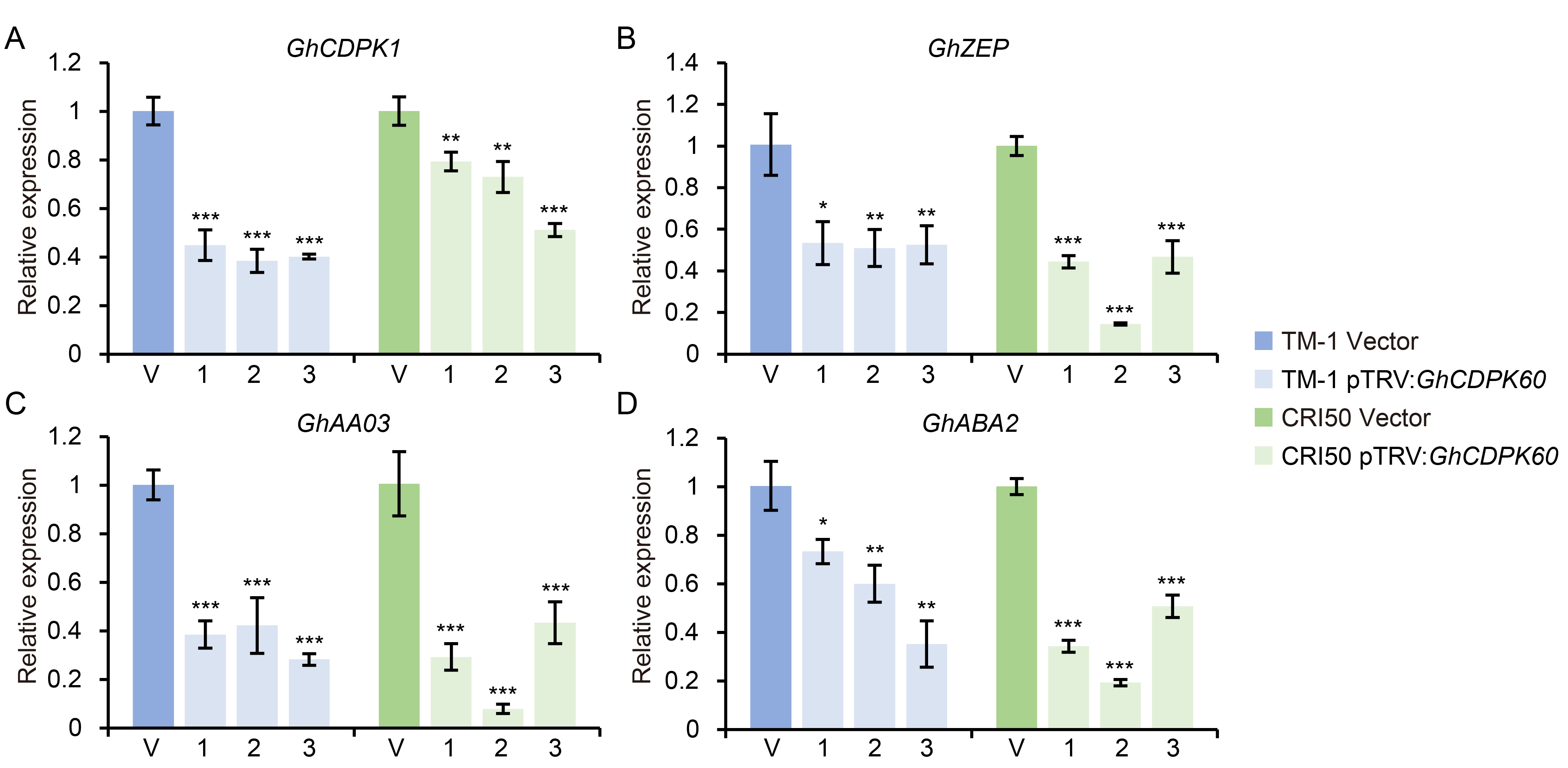

Supplement: Supplementary Figure 6 — Relative expression of four stress-related genes, including GhCDPK1 (A), GhZEP (B), GhAAO3 (C), and GhABA2 (D) in control and GhCDPK60-silenced plants after drought treatment. V, lines infected with empty vector. 1-3 indicated three individual plants. Statistically significant differences compared to control (lines infected with empty vector). Values represent the mean ± SE from three biological replicates. *P< 0.05, **P< 0.01 and ***P< 0.001 by Student’s t test. [file Image_6.jpg]
