# Supplementary material for: GhCDPK60 positively regulates drought stress tolerance in both transgenic Arabidopsis and cotton by regulating proline content and ROS level
Source: Front Plant Sci. 2022 Dec 1;13:1072584. doi: 10.3389/fpls.2022.1072584 (PMC9751749; doi:10.3389/fpls.2022.1072584)
Supplement: Supplementary file 7 [file Table_1.docx]

Supplementary Tables for

**GhCDPK60 positively regulates drought stress tolerance in both transgenic *Arabidopsis* and cotton by regulating proline content and ROS level**

**Supplementary Table S1.** *GhCDPKs* Identified in *G. hirsutum* Genomes

**Supplementary Table S2.** Primer pairs used in this study

**Supplementary Table S3.** Analysis of *GhCDPK60* promoter

**Supplementary Table S1 *GhCDPKs* Identified in *G. hirsutum* Genomes**

| **Gene ID** | **Name** | **Length (aa)** | **Chr.** | **MW (kDa)** | **Isoelectric Point** |
| --- | --- | --- | --- | --- | --- |
| *Ghir_A01G007100.1* | *GhCDPK1* | 537 | A01 | 60.545 | 6.470 |
| *Ghir_A01G012430.1* | *GhCDPK2* | 569 | A01 | 64.131 | 6.472 |
| *Ghir_A02G001700.1* | *GhCDPK3* | 395 | A02 | 45.094 | 4.844 |
| *Ghir_A02G006230.1* | *GhCDPK4* | 511 | A02 | 57.149 | 5.507 |
| *Ghir_A02G011170.1* | *GhCDPK5* | 519 | A02 | 58.607 | 5.768 |
| *Ghir_A02G012330.1* | *GhCDPK6* | 535 | A02 | 60.708 | 6.473 |
| *Ghir_A02G018670.1* | *GhCDPK7* | 541 | A02 | 61.343 | 9.256 |
| *Ghir_A03G018780.1* | *GhCDPK8* | 568 | A03 | 63.624 | 6.117 |
| *Ghir_A04G001660.1* | *GhCDPK9* | 524 | A04 | 58.832 | 6.359 |
| *Ghir_A04G005530.1* | *GhCDPK10* | 648 | A04 | 71.678 | 5.128 |
| *Ghir_A04G005600.1* | *GhCDPK11* | 572 | A04 | 63.564 | 5.159 |
| *Ghir_A04G009910.1* | *GhCDPK12* | 527 | A04 | 59.400 | 6.393 |
| *Ghir_A04G012050.1* | *GhCDPK13* | 552 | A04 | 61.901 | 5.500 |
| *Ghir_A05G007640.1* | *GhCDPK14* | 532 | A05 | 59.526 | 5.314 |
| *Ghir_A05G018710.1* | *GhCDPK15* | 523 | A05 | 58.933 | 5.930 |
| *Ghir_A05G028110.1* | *GhCDPK16* | 502 | A05 | 56.473 | 5.531 |
| *Ghir_A05G033000.1* | *GhCDPK17* | 534 | A05 | 60.707 | 7.095 |
| *Ghir_A06G000080.1* | *GhCDPK18* | 526 | A06 | 59.249 | 6.339 |
| *Ghir_A06G022050.1* | *GhCDPK19* | 527 | A06 | 59.353 | 5.082 |
| *Ghir_A07G012870.1* | *GhCDPK20* | 512 | A07 | 57.321 | 5.498 |
| *Ghir_A07G013130.1* | *GhCDPK21* | 464 | A07 | 52.312 | 6.755 |
| *Ghir_A08G001480.1* | *GhCDPK22* | 538 | A08 | 60.501 | 5.241 |
| *Ghir_A09G011860.1* | *GhCDPK23* | 523 | A09 | 58.559 | 5.898 |
| *Ghir_A09G012230.1* | *GhCDPK24* | 415 | A09 | 46.647 | 8.262 |
| *Ghir_A09G013220.1* | *GhCDPK25* | 423 | A09 | 47.730 | 8.465 |
| *Ghir_A09G014160.1* | *GhCDPK26* | 610 | A09 | 68.253 | 4.846 |
| *Ghir_A10G000830.1* | *GhCDPK27* | 552 | A10 | 62.216 | 6.532 |
| *Ghir_A10G009700.1* | *GhCDPK28* | 552 | A10 | 61.930 | 8.627 |
| *Ghir_A10G013610.1* | *GhCDPK29* | 537 | A10 | 61.318 | 6.030 |
| *Ghir_A10G020050.1* | *GhCDPK30* | 400 | A10 | 44.817 | 7.135 |
| *Ghir_A11G002410.1* | *GhCDPK31* | 530 | A11 | 60.401 | 6.869 |
| *Ghir_A11G003380.1* | *GhCDPK32* | 550 | A11 | 62.333 | 6.525 |
| *Ghir_A11G018500.1* | *GhCDPK33* | 503 | A11 | 56.996 | 9.384 |
| *Ghir_A11G035540.1* | *GhCDPK34* | 527 | A11 | 59.391 | 6.520 |
| *Ghir_A12G001310.1* | *GhCDPK35* | 560 | A12 | 62.572 | 5.809 |
| *Ghir_A12G024600.1* | *GhCDPK36* | 534 | A12 | 60.274 | 6.280 |
| *Ghir_A13G000100.1* | *GhCDPK37* | 579 | A13 | 64.654 | 6.054 |
| *Ghir_A13G006450.1* | *GhCDPK38* | 523 | A13 | 58.563 | 6.975 |
| *Ghir_A13G006470.1* | *GhCDPK39* | 487 | A13 | 54.631 | 4.855 |
| *Ghir_A13G014410.1* | *GhCDPK40* | 536 | A13 | 60.369 | 6.277 |
| *Ghir_A13G023110.1* | *GhCDPK41* | 377 | A13 | 42.707 | 5.229 |
| *Ghir_D01G007400.1* | *GhCDPK42* | 537 | D01 | 60.469 | 6.564 |
| *Ghir_D01G013420.1* | *GhCDPK43* | 591 | D01 | 66.347 | 5.181 |
| *Ghir_D02G001790.1* | *GhCDPK44* | 532 | D02 | 60.755 | 5.355 |
| *Ghir_D02G006620.1* | *GhCDPK45* | 508 | D02 | 56.847 | 5.504 |
| *Ghir_D02G020140.1* | *GhCDPK46* | 568 | D02 | 63.550 | 6.020 |
| *Ghir_D03G000930.1* | *GhCDPK47* | 480 | D03 | 54.767 | 7.983 |
| *Ghir_D03G007610.1* | *GhCDPK48* | 550 | D03 | 62.297 | 7.067 |
| *Ghir_D04G010070.1* | *GhCDPK49* | 401 | D04 | 45.269 | 4.452 |
| *Ghir_D04G010120.1* | *GhCDPK50* | 593 | D04 | 65.991 | 5.418 |
| *Ghir_D04G014130.1* | *GhCDPK51* | 368 | D04 | 41.626 | 9.037 |
| *Ghir_D04G016440.1* | *GhCDPK52* | 552 | D04 | 61.953 | 5.128 |
| *Ghir_D05G007720.1* | *GhCDPK53* | 536 | D05 | 60.097 | 5.925 |
| *Ghir_D05G018740.1* | *GhCDPK54* | 517 | D05 | 58.334 | 5.760 |
| *Ghir_D05G028130.1* | *GhCDPK55* | 513 | D05 | 57.624 | 5.543 |
| *Ghir_D05G033690.1* | *GhCDPK56* | 517 | D05 | 58.879 | 7.421 |
| *Ghir_D05G037820.1* | *GhCDPK57* | 403 | D05 | 45.639 | 5.155 |
| *Ghir_D06G023100.1* | *GhCDPK58* | 527 | D06 | 59.329 | 5.232 |
| *Ghir_D07G013040.1* | *GhCDPK59* | 513 | D07 | 57.319 | 5.143 |
| *Ghir_D07G013310.1* | *GhCDPK60* | 529 | D07 | 59.438 | 7.402 |
| *Ghir_D09G011400.1* | *GhCDPK61* | 457 | D09 | 50.309 | 6.442 |
| *Ghir_D09G011730.1* | *GhCDPK62* | 531 | D09 | 59.855 | 6.523 |
| *Ghir_D09G011910.1* | *GhCDPK63* | 576 | D09 | 64.395 | 9.135 |
| *Ghir_D09G012710.1* | *GhCDPK64* | 531 | D09 | 60.234 | 6.756 |
| *Ghir_D09G013620.1* | *GhCDPK65* | 609 | D09 | 68.095 | 5.107 |
| *Ghir_D10G001600.1* | *GhCDPK66* | 551 | D10 | 62.026 | 6.165 |
| *Ghir_D10G009490.1* | *GhCDPK67* | 535 | D10 | 61.001 | 8.962 |
| *Ghir_D10G013990.1* | *GhCDPK68* | 533 | D10 | 60.797 | 5.662 |
| *Ghir_D10G021750.1* | *GhCDPK69* | 583 | D10 | 64.906 | 5.139 |
| *Ghir_D11G002390.1* | *GhCDPK70* | 530 | D11 | 60.361 | 6.564 |
| *Ghir_D11G003360.1* | *GhCDPK71* | 550 | D11 | 62.319 | 6.525 |
| *Ghir_D11G018640.1* | *GhCDPK72* | 554 | D11 | 62.798 | 9.481 |
| *Ghir_D11G036410.1* | *GhCDPK73* | 527 | D11 | 59.305 | 6.522 |
| *Ghir_D12G001340.1* | *GhCDPK74* | 574 | D12 | 64.468 | 6.152 |
| *Ghir_D12G024580.1* | *GhCDPK75* | 534 | D12 | 60.249 | 6.388 |
| *Ghir_D13G000300.1* | *GhCDPK76* | 472 | D13 | 52.578 | 7.171 |
| *Ghir_D13G005790.1* | *GhCDPK77* | 523 | D13 | 58.408 | 6.410 |
| *Ghir_D13G005800.1* | *GhCDPK78* | 528 | D13 | 58.972 | 4.980 |
| *Ghir_D13G015090.1* | *GhCDPK79* | 536 | D13 | 60.426 | 6.374 |
| *Ghir_D13G023730.1* | *GhCDPK80* | 527 | D13 | 58.800 | 6.426 |

**Supplementary Table S2 Primer pairs used in this study**

| Usage | Primer Name | Sequence (5ʹ - 3ʹ) |
| --- | --- | --- |
| Construct for overexpression | pBI121-GhCDPK60-F | AGAACACGGGGGACTCTAGATGGATCAGAGCGAGTTGTTGG |
|  | pBI121-GhCDPK60-R | GATCGGGGAAATTCGAGCTCTGCAACTCACTCATCACAAATCT |
| Construct for localization analysis | 1305-35S-GhCDPK-GFP-F | GCCCAGATCAACTAGTATGGGAAATTGCTGTGCTACC |
|  | 1305-35S-GhCDPK-GFP-R | TCGAGACGTCTCTAGAAGTGGCCACTACCTGCATCA |
| Construct for VIGS analysis | pTRV-GhCDPK60-F | AGTGGTCTCTGTCCAGTCCTGGAGCTCAAGGCAGGATTGC |
|  | pTRV-GhCDPK60-R | GGTCTCAGCAGACCACAAGTCCAATCGGTACCGGCTTTCA |
| RT-qPCR | qRT-GhCDPK60-F | ACACCAAACAACGAGGCAAA |
|  | qRT-GhCDPK60-R | GAGACCCATCACCATCACCA |
|  | qRT-GhACT4-F | ATCCTCCGTCTTGACCTTG |
|  | qRT-GhACT4-R | TGTCCGTCAGGCAACTCAT |
|  | qRT-AtUBQ7-F | CGTTGCCTCAAAAGATGCAGATC |
|  | qRT-AtUBQ7-R | ACATTGTCGATGGTGTCGGATG |
|  | qRT-AtCAT3-F | CGCCCCATTCTACACCACAA |
|  | qRT-AtCAT3-R | CCGGACCTCTTTCTCCGATG |
|  | qRT-AtNXH1-F | ACCCCAAAATCCATACATATCCC |
|  | qRT-AtNXH1-R | CCACGACCTCCAAAGACGG |
|  | qRT-AtRD29A-F | ACGTTTGCTCCAAGTGGTGA |
|  | qRT-AtRD29A-R | CCTCCAACGTTATCGGGGTC |
|  | qRT-AtRD29B-F | GGCGGGCAAAGCGAG |
|  | qRT-AtRD29B-R | TGCCCGTAAGCAGTAACAGATC |
|  | qRT-AtDREB2A-F | GACCTAAATGGCGACGATGT |
|  | qRT-AtDREB2A-R | GCGGATCAAAACCACTTTGT |
|  | qRT-GhCDPK1-F | GGAGCCCATACTATGTTGC |
|  | qRT-GhCDPK1-R | GCCTTGCTCGCTTTCAG |
|  | qRT-GhABA2-F | GCTCATTGCTTCCAGTCTCCA |
|  | qRT-GhABA2-R | TTTTCAGTCAACCCTACCACAGC |
|  | qRT-GhAAO3-F | GCAAACCCAAACTACACCCACA |
|  | qRT-GhAAO3-R | GCCACAGCCTCCTTCACCA |
|  | qRT-GhZEP-F | CTTCCCGCAAACAACATTCG |
|  | qRT-GhZEP-R | GCCTTCCTCCTGCCATTATCA |

**Supplementary Table S3 Analysis of *GhCDPK60* promoter**

| Element | Sequence | Number | Function |
| --- | --- | --- | --- |
| ABRE | ACGTG | 2 | Cis-acting elements involved in the ABA reaction |
| Box 4 | ATTAAT | 5 | Conserved sequences involved in photosynthesis |
| CAAT-box | CCAAT | 48 | Cis-acting elements in promoter and enhancer regions |
| CGTCA-motif | CGTCA | 1 | Cis-acting regulatory elements involved in MeJA reactivity |
| G-box | CACGAC | 2 | Cis-acting regulatory elements involved in photosynthesis |
| GCN4_motif | TGAGTCA | 1 | Cis-regulatory elements involved in endosperm expression |
| TATA-box | TATTTAAA | 40 | The core promoter element located -30 bp from transcription initiation |
| WUN-motif | AAATTTCCT | 2 | Wound response element |
| MBS | CAACTG | 1 | MYB binding sites involved in drought induction |
